# Two-stage isolated AC/DC converter and its compound control strategy

**DOI:** 10.1371/journal.pone.0275056

**Published:** 2022-09-22

**Authors:** Kai Zhou, Da Teng, Chengxiang Yuan

**Affiliations:** Engineering Research Center of Automotive Electronics Drive Control and System Integration, Ministry of Education, Harbin University of Science and Technology, Harbin, Heilongjiang, China; Karlstad University: Karlstads Universitet, SWEDEN

## Abstract

With the continued development of the new energy vehicle industry, two-stage isolated AC/DC converters are widely used because of their simple topology and easy control characteristics. In this study, we investigate the front-stage Buck power factor correction (PFC) converter and rear-stage full-bridge converter. The main circuit design and component selection were completed through a detailed analysis of the circuit characteristics. In terms of the control strategy, the front-stage adopting PI control and parameter adaptive terminal sliding mode control strategy were proposed for the rear-stage full-bridge converter. This new compound control strategy ensures an optimal regulation of the system under different operating conditions. Simulation analysis verified the correctness of the system topology and control strategy. Based on an analysis of the main parameters of the system, a low-power experimental prototype was trial-produced. The experimental results show that under the same load switching conditions, the parameter-adaptive terminal sliding mode control enhanced faster dynamic regulation and stronger robustness than the conventional PI control. The study is also a good reference in terms of engineering work.

## 1 Introduction

With the development of power electronics technology, high efficiency, high power density, and wide voltage range of AC/DC converter using two-stage circuit structure have become the industry research hotspot.

The front stage of the AC/DC converter is a power factor correction circuit, which can improve the power factor and reduce grid-side current harmonics. Its performance affects the utilization of grid energy and control effect of the rear-stage DC/DC converter. Current research on PFC circuits mainly focuses on Boost and its improved circuits. However, traditional Boost PFC circuits have a high output voltage, which has great voltage stress on the rear-stage components and is not conducive to rear-stage circuit design. Improved circuits also have limitations such as complex drive design and low overall efficiency [1]. The Buck PFC circuit has a low-voltage stress requirement on the component and strong ability to control the input and output currents. It is particularly suitable for high-voltage input and low-voltage operation of the load; however, owing to its structural limitations, the input current has a dead-time. Scholars have proposed several solutions to this problem. For example, in [2], the circuit operates in Buck-Boost mode at dead-time by adding auxiliary switches and diodes, and a nonlinear control strategy was used to achieve power-factor correction. In [3], an improved Buck PFC circuit was proposed, which works in Buck-Boost mode when the input voltage is lower than the output voltage. In [4], the Buck converter was combined with Flyback converter in parallel, which eliminated the input current dead-time and reduced the current harmonics. In [5], a Buck circuit with an active buffer was proposed to reduce the input current dead-time. In [6], a series-capacitor based interleaved Buck PFC converter was proposed, which reduces the current dead time through extremely low output voltage and achieves high power factor. The circuit structure and control strategy used in the aforementioned studies are still relatively complex. Combined with the design requirements of a two-stage low-power experimental prototype, this study investigated the Buck PFC circuit.

The rear-stage of an AC/DC converter often uses an isolated DC/DC converter. Full-bridge converters have been widely used in many fields owing to their high power density, high voltage conversion ratio, and low switching loss [7,8]. Compared to other converter topologies, the full-bridge converter can realize zero-voltage switching by the resonance of its own parasitic parameters, which greatly reduces the switching loss of components, and improves the efficiency of the circuit. Therefore, this study considers the full-bridge converter as the research object of the rear-stage main circuit.

From the perspective of the full-bridge converter control strategy, traditional PID control has many advantages, such as simple design, convenient application, and easy hardware implementation [9]. However, there are also problems, such as slow start speed and long adjustment time. Thus, various new control strategies have been proposed. For example, in [10], a fuzzy control was combined with PI control to overcome the unfavorable factors such as variable parameters and nonlinearity of the system to some extent. in [11], a BP neural network was introduced into the generation process of PID parameters, and the PID parameters were adjusted in real time based on the circuit state to optimize the PID parameters. However, neural networks should be driven by big data. This is quite different from the circuit characteristics of the full-bridge converter. Therefore, the training effect is difficult to guarantee.

In recent years, sliding mode control has been widely applied to DC/DC converters due to its easy design and robustness. Currently, the studies on sliding mode control strategy for DC/DC converter topologies are mainly focused on Buck circuit. In [12], a method for designing sliding mode coefficients was proposed, which is based on the circuit parameters and design index of Buck converter. In [13], a fractional-order sliding mode control strategy was proposed, which not only reduces the sensitivity of the system to mismatched interference, but also improves the transient performance of the system. In addition, adaptive hysteresis sliding mode control method [14] and second-order sliding mode control method [15] have also achieved favorable control effects. However, the control strategy designed for the Buck circuit cannot be directly applied to the full-bridge converter, which requires a new mathematical model and an equivalent control law.

Some solutions have been proposed by scholars for the sliding mode control strategy of full-bridge converters. In [16], a PWM full-order sliding mode control strategy for full-bridge converters was proposed, which makes the full-bridge converter operate in the fixed frequency state and shows strong robustness when the external working state changes. In [17], the backstepping method was combined with sliding mode control theory, which overcomes the strong dependence of the traditional backstepping method on the system model and shows strong resistance to input and load disturbances. In [18], a method combining adaptive fuzzy control and sliding mode control was adopted, which significantly improves the system performance. In [19], a double integral indirect sliding mode control strategy was proposed to effectively eliminate the state error in the output and improve the dynamic performance of the full-bridge circuit. The above strategies based on the sliding mode control theory have the characteristics of strong robustness. However, there are still problems in that parameters depend strongly on the system, and different parameter settings have a significant influence on the system control effect. Therefore, a parameter-adaptive control algorithm with a fast-approaching speed is required to ensure that the system has a better dynamic performance.

Based on the above discussion, a two-stage isolated AC/DC converter was designed in this study, and a compound control strategy with power factor correction, strong dynamic recovery performance and parameter adaption was proposed. The main contributions of this paper are as follows:

1. The front-stage PFC circuit adopts a Buck circuit with an LC filter, and makes it operate in the discontinuous capacitor voltage mode (DCVM). Through detailed analysis of the circuit principle and reasonable parameter selection, the input current dead-time is eliminated and the power factor correction is realized.
2. The equivalent mathematical model of the full-bridge circuit is established based on the state-space averaging method, which lays the foundation for the controller design.
3. A parameter adaptive terminal sliding mode control strategy for full-bridge converter is proposed. The strategy has strong anti-interference capability and the parameters γ can be adjusted in real time according to the system state to ensure the optimal control effect of the system under different operating conditions.

This paper is organized as follows. Section 2 introduces the analysis and design of the front-stage Buck PFC circuit. Section 3 presents the analysis and design of the rear-stage full-bridge DC/DC converter. Section 4 introduces the control strategies of the front and rear stages respectively, and shows the simulation results. Section 5 shows and analyzes the experimental results. Finally, Section 6 summarizes the conclusions.

## 2 Analysis and design of front-stage Buck PFC circuit

### 2.1 Analysis of operating characteristics of Buck PFC circuit in DCVM mode

**Fig 1:**
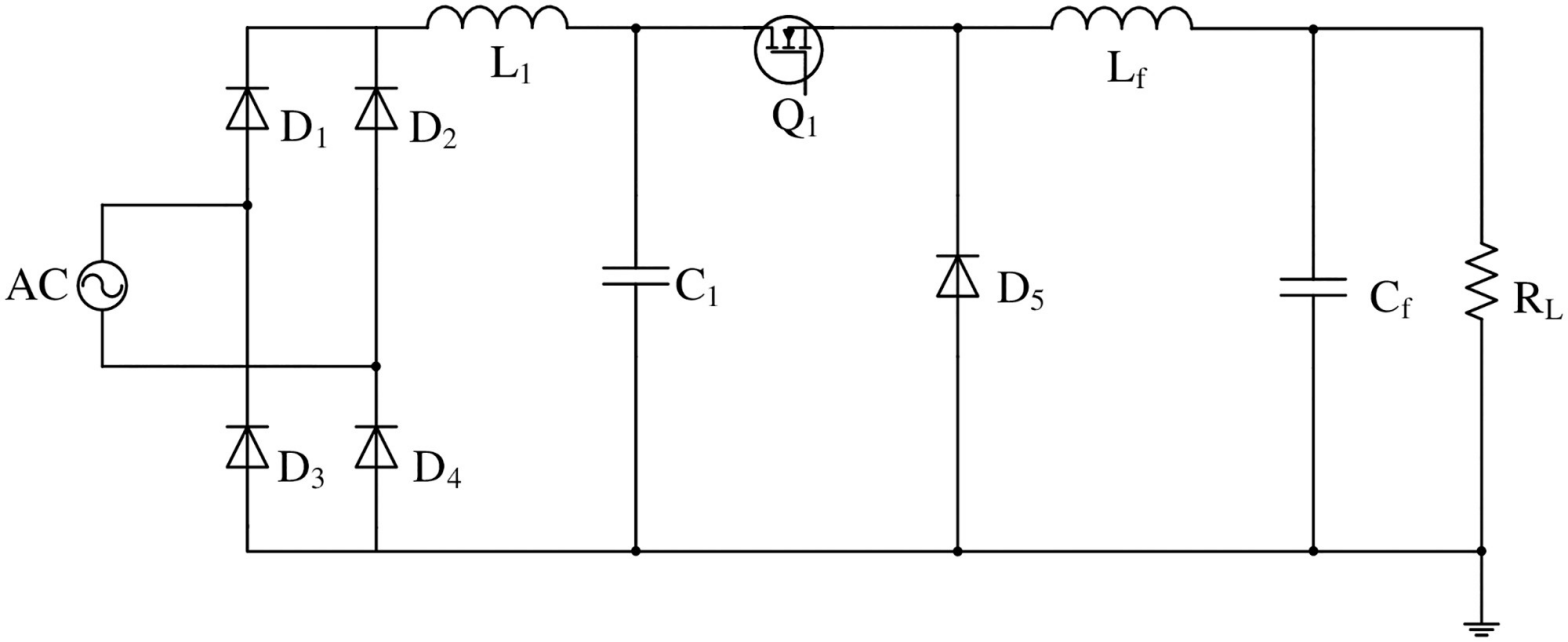
Buck PFC circuit operating in DCVM mode.

**Fig 2:**
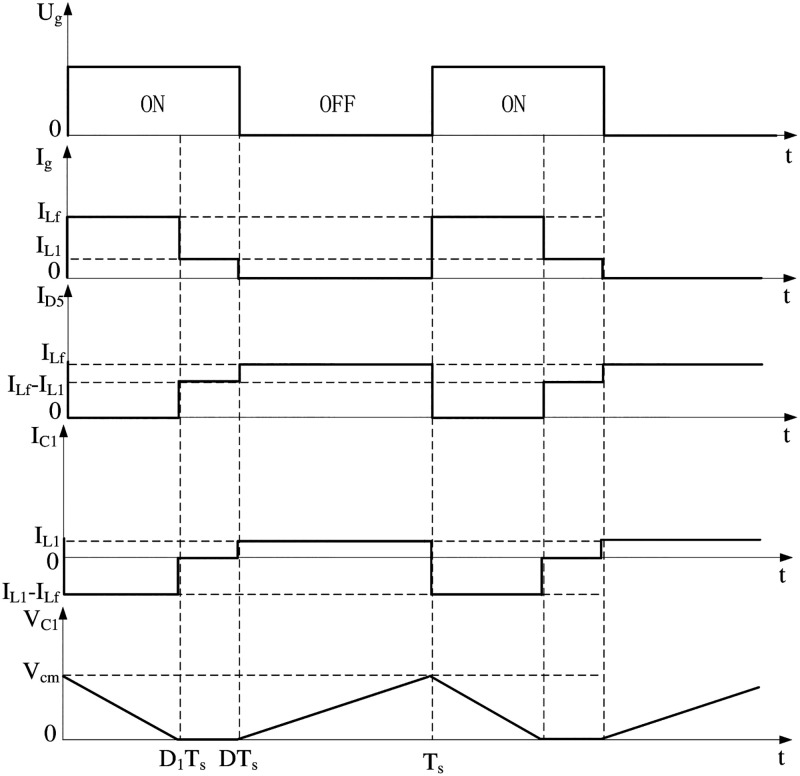
Working waveforms of Buck PFC circuit in DCVM mode.

The front stage uses a Buck PFC circuit operating in discontinuous capacitor voltage mode [20], as shown in (Fig 1). An LC filter circuit was added to the input side of the conventional Buck PFC circuit. The value of inductor L_1_ is sufficiently large to ensure that the current flowing through L_1_ is always continuous during the switching cycle. The value of capacitor C_1_ is sufficiently low to ensure that the voltage across MOSFET Q_1_ can drop to zero when Q_1_ turns on.

(Fig 2) shows a typical waveform of the Buck PFC circuit working in the DCVM mode. A complete switching cycle consists of three operating modes, which can be briefly summarized as capacitor C_1_ discharge mode, diode D_5_ freewheeling mode, and capacitor C_1_ charging mode.

From (Fig 2), when *T = T*_s_, the voltage across capacitor C_1_ reaches its maximum, and the peak voltage is

$$V_{\text{cm}}=\frac{I_{\text{L1}}}{C_{\text{1}}}\text{(1-}D\text{)}T_{\text{s}}$$ (1)

where *V*_cm_ is the peak voltage across capacitor C_1_, *I*_L1_ is the current of inductor L_1_ and *D* is the duty cycle.

According to the principle of volt-second balance, the average voltage across inductors L_1_ and L_f_ were both 0 V at steady state, thus the expression can be obtained:

$$V_{\text{rec}}=\frac{\text{(1-}D\text{+}D_{\text{1}}\text{)}V_{\text{cm}}}{\text{2}}$$ (2)

$$V_{\text{o}}\;=\frac{D_{\text{1}}V_{\text{cm}}}{\text{2}}$$ (3)

where *V*_rec_ is the rectified voltage on the input side, *V*_o_ is the output voltage, and *D*_1_ is the proportionality coefficient.

From Eqs (1)–(3), we obtain:

$$R_{\text{1}}=\frac{T_{\text{s}}}{\text{2}C_{\text{1}}}{\text{(1-}D\text{)}}^{\text{2}}\frac{V_{\text{rec}}}{V_{\text{rec}}\text{-}V_{\text{o}}}$$ (4)

where *R*_1_ is the equivalent resistance viewed from the input side.

For convenience, the derivation of the subsequent formulas is based on the following assumptions:

1. The input voltage is a sine wave after the rectification. The input voltage is expressed as *V*_in_=*V*_1_sin*w*_L_*t*, where *w*_L_=2π/*T*, *V*_1_ is the input voltage peak, *T* is the power frequency voltage period, and *T* >> *T*_s_.
2. The filter capacitor C_f_ is sufficiently large to ensure that the output voltage can be regarded as a constant within half a power-frequency period.
3. The input side inductance L_1_ and output side inductance L_f_ are sufficiently large to ensure that the current flowing through them in one switching cycle can be regarded as a constant.

Based on the above assumptions, the circuit operates only when the input voltage is greater than the output voltage. There is an edge conduction time *t*_edge_, which can be expressed as:

$$t_{\text{edge}}=\frac{\text{1}}{w_{\text{L}}}\text{arcsin}\frac{V_{\text{o}}}{V_{\text{1}}}=\frac{\text{1}}{w_{\text{L}}}\text{arcsin}M_{\text{s}}$$ (5)

where *M*_s_=*V*_o_/*V*_1_ is the ratio of the output voltage to the sinusoidal input voltage peak.

Therefore, it can be inferred that the circuit can operate normally only when $t\in (t_{\text{edge}},T\text{/2}-t_{\text{edge}})$ during half a power frequency period, and the primary-side rectifier diodes D_1_ and D_4_ can turn on normally.

According to the law of energy conservation and conversion efficiency, the relationship between the output energy and input energy is *W*_2_ = *ηW*_1_, which can be obtained as

$$\frac{KM_{\text{s}}^{2}{\text{(1-}D\text{)}}^{\text{2}}}{\text{4}}=\eta \text{[}\frac{1}{2}\text{-}\frac{\text{1}}{\pi }\text{arcsin}M_{\text{s}}\text{-}\frac{M_{\text{s}}\sqrt{\text{1-}M_{\text{s}}^{2}}}{\pi }\text{]}$$ (6)

where *K* is the parameter, *K*=2*T*_*s*_ / (*R*_L_*C*_1_).

**Fig 3:**
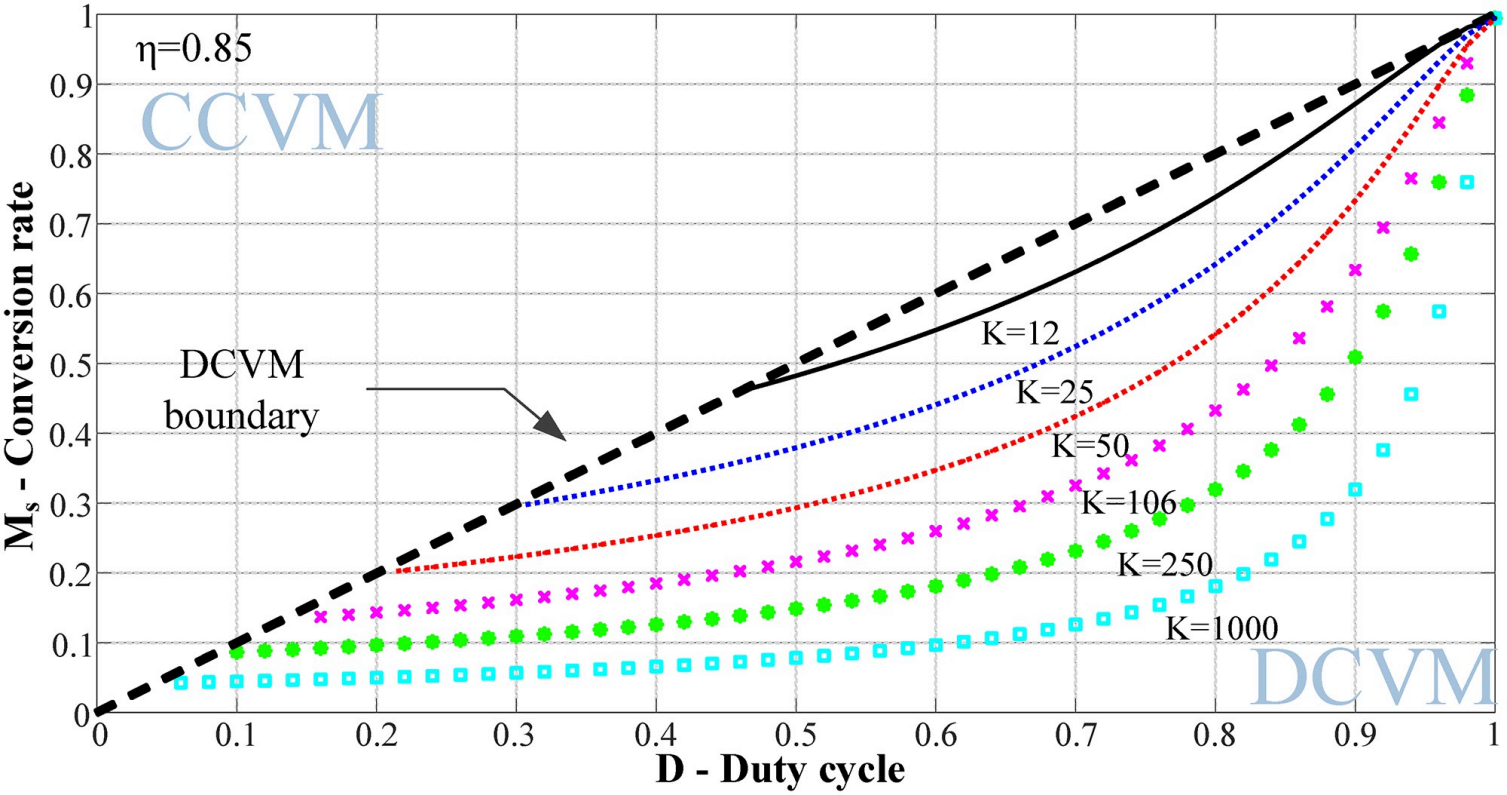
*M*_s_-*D* curve at efficiency *η* =0.85.

Eq (6) contains four variables *K*, *D*, *η*, and *M*_s_, where *K*, *D*, and *η* can be regarded as independent variables and *M*_s_ can be regarded as dependent variables. Different values of *M*_s_ can be obtained by assigning different values to *K*, *D*, and *η*. Thus, the relationship curve of the voltage conversion rate *M*_s_ with the duty cycle *D* at different *K* values at a certain efficiency can be drawn.

(Fig 3) shows the *M*_s_-*D* curve when *η* =0.85. The black double dashed line is the system boundary curve indicating whether the circuit can operate in the DCVM mode. When parameter *K* increases, the curve moves downward, and the effective duty cycle adjustment range of the circuit increases.

However, different values of *K* affect the voltage stress of the MOSFET. The maximum time of the MOSFET voltage stress was the time of the input voltage peak. At this time, the voltage across the MOSFET was.


$$V_{\text{ds}}=\frac{\text{2(}V_{\text{1}}\text{-}V_{\text{o}}\text{)}}{\text{1-}D}$$ (7)

The ratio of the voltage borne by the MOSFET to the peak input sinusoidal voltage is defined as *K*_s_=*V*_ds_ / *V*_1_. Substituting the *K*_s_ expression into Eq (7), we obtain

$$K_{\text{s}}=\frac{\text{2(1-}M_{\text{s}}\text{)}}{\text{1-}D}$$ (8)

Where *D* is eliminated by combining Eqs (6) and (8), the expressions for *K*, *M*_s_, *K*_s_, and *η* can be obtained:

$$K{\text{(}M_{\text{s}}\text{-}M_{\text{s}}^{2}\text{)}}^{\text{2}}\text{-}K_{\text{s}}^{2}\eta \text{[}\frac{1}{2}\text{-}\frac{1}{\pi }\text{arcsin}M_{\text{s}}\text{-}\frac{M_{\text{s}}\sqrt{\text{1-}M_{s}^{2}}}{\pi }\text{]=0}$$ (9)

**Fig 4:**
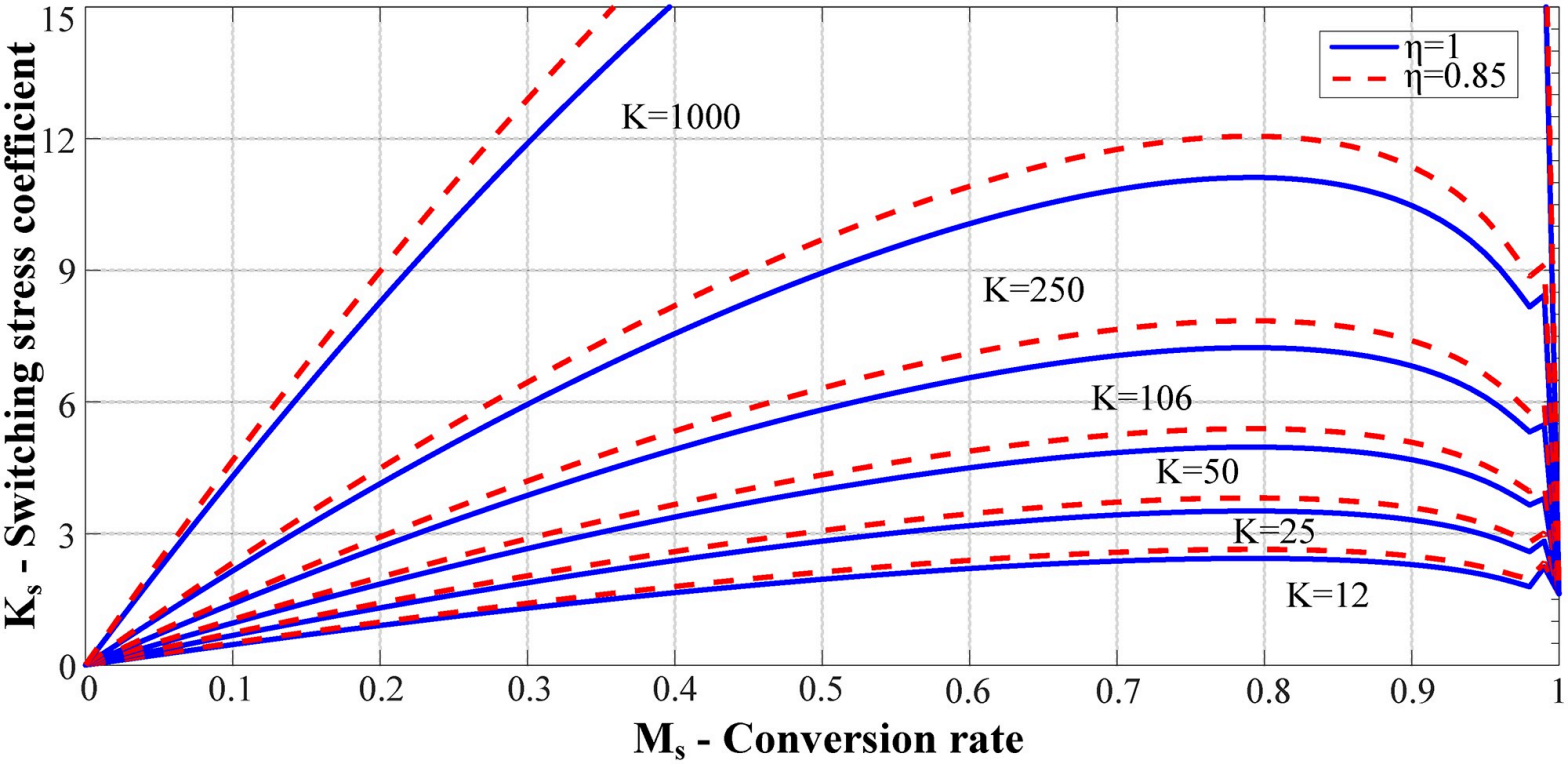
*M*_s_-*K*_s_ curve under different parameter *K* and *η*.

In Eq (9), *K* and *η* are considered as constants, while *M*_s_ and *K*_s_ are considered as independent and dependent variables, respectively. (Fig 4) shows the curves of the relationship between *M*_s_ and *K*_s_ corresponding to different parameters *K* and efficiency *η*.

(Fig 4) shows that for the same conversion ratio *M*_s_, the larger the value of *K*, the higher is the voltage stress borne by the MOSFET. As the efficiency of the circuit decreased, the voltage stress increased. To reduce the voltage stress, the value of parameter *K* should be as small as possible. However, considering the *M*_s_-*D* curve, the value of *K* should be as large as possible to ensure that the circuit has a larger range of load regulation. Therefore, when determining parameter *K*, it is necessary to comprehensively consider the MOSFET voltage stress and duty cycle regulation range to determine the optimal value of parameter *K*.

### 2.2 Determination of Buck PFC circuit parameters in DCVM mode

The specific design parameters of the circuit are listed in Table 1 for the design requirements of a small power converter.

**Table 1:** Design parameters of Buck PFC circuit.

Design parameters	Reference values
Input voltage	90-264 VAC
Output voltage	24 VDC
Rated power	100 W
Output voltage ripple	≤5%
Input current THD	IEC61000-3-2 Class C
Input side PF	≥95%

Referring to the design parameter requirements in Table 1, we can calculate the parameters of the Buck PFC circuit operate in DCVM mode. Considering that the input voltage range is 90-264 VAC, that is, there is a limit to the value range of *M*_s_. Substituting it into Eq (5), the value interval of *M*_s_ can be obtained as

$$0.064\leq M_{s}\leq 0.189$$ (10)

Additionally, it is important to consider the circuit performance when the RMS of the input voltage is 220V, that is *M*_s_ = 0.077.

Here, *M*_s_ is 0.064, 0.077, and 0.189, respectively. The corresponding duty-cycle values under different *K* values and whether the circuit can operate in the DCVM mode are listed in Table 2.

**Table 2:** Analysis of circuit operating characteristics under different *K* values at *η* =0.85.

*K*	1000	900	800	700	600	500	400	350	300
**Duty cycle *D* when *M***_***s***_ **=0.064**	0.38	0.35	0.31	0.26	0.20	0.13	0.02	-0.04	-0.13
**Duty cycle *D* when *M***_***s***_ **=0.077**	0.49	0.46	0.43	0.39	0.34	0.28	0.19	0.14	0.07
**Duty cycle *D* when *M***_***s***_ **=0.189**	0.81	0.79	0.78	0.77	0.75	0.73	0.69	0.67	0.65
**Whether it can operate** **in DCVM**	Y	Y	Y	Y	Y	Y	N	N	N

From Table 2, to ensure that the circuit operates in the DCVM mode, the value of *K* should be greater than or equal to 500. Combined with the *M*_s_-*K*_s_ relationship curve, for the same voltage conversion ratio *M*_s_, reducing the value of parameter *K* can reduce the voltage stress on the MOSFET. Therefore, after a comprehensive consideration, *K* = 500.

Substituting the switching frequency *f*_s_ = 100kHz and equivalent load *R*_L_ = 6 Ω into *K*=2*T*_s_/(*R*_L_*C*_1_), we calculated *C*_1_ = 6.67 nF.

Inductor *L*_1_ should ensure that the current flowing through it in a switching cycle is continuous. Basically, the resonant period of the resonant current flowing through *L*_1_ is significantly larger than the switching period of the MOSFET. This satisfies the following formula:

$$2\pi \sqrt{L_{\text{1}}C_{\text{1}}}\gg \text{(1}-D\text{)}T_{\text{s}}$$ (11)

However, the value of *L*_1_ is not too large, thus inductance *L*_1_ can be ignored from the input side. Therefore, this value should satisfy the following requirements:

$$R_{\text{1}}\gg \frac{2\pi L_{\text{1}}}{T}$$ (12)

Combining Eqs (4), (11) and (12), the value range of *L*_1_ can be obtained as

$$\frac{{\text{(1}-D\text{)}}^{\text{2}}T_{\text{s}}^{2}}{{4\pi }^{\text{2}}C_{\text{1}}}\ll L_{\text{1}}\ll \frac{T_{\text{s}}TV_{\text{rec}}{\text{(1}-D\text{)}}^{\text{2}}}{4\pi C_{\text{1}}\text{(}V_{\text{rec}}-V_{\text{o}}\text{)}}$$ (13)

By substituting into the circuit parameters when *V*_rms_ = 220 V and *K* = 500, the value range of *L*_1_ can be calculated. Based on a comprehensive consideration, *L*_1_ = 3.2 mH.

Inductance *L*_f_ can be determined using the derivation method of *L*_1_. When the MOSFET is turned on, the current flowing through inductor *L*_f_ is guaranteed to be continuous in one switching cycle, which can be obtained as

$$2\pi \sqrt{\frac{L_{\text{1}}L_{\text{f}}C_{\text{1}}}{L_{\text{1}}\text{+}L_{\text{f}}}}\gg DT_{\text{s}}$$ (14)

Further:

$$L_{\text{f}}\gg \frac{D^{\text{2}}T_{\text{s}}^{2}L_{\text{1}}}{{\text{4π}}^{\text{2}}L_{\text{1}}C_{\text{1}}\text{-}D^{\text{2}}T_{\text{s}}}$$ (15)

Eq (15) describes only the lower limit of inductance *L*_f_. The determination of *L*_f_ also should comprehensively consider factors such as inductor current ripple and finally select *L*_f_ = 250 μH. The determination of capacitor *C*_f_ should comprehensively consider the output voltage ripple and the voltage holding time, and finally select *C*_f_ = 20 mF.

## 3 Analysis and design of rear-stage full-bridge DC/DC converter

### 3.1 Analysis of operating characteristics of Full-Bridge converter with clamping diode

**Fig 5:**
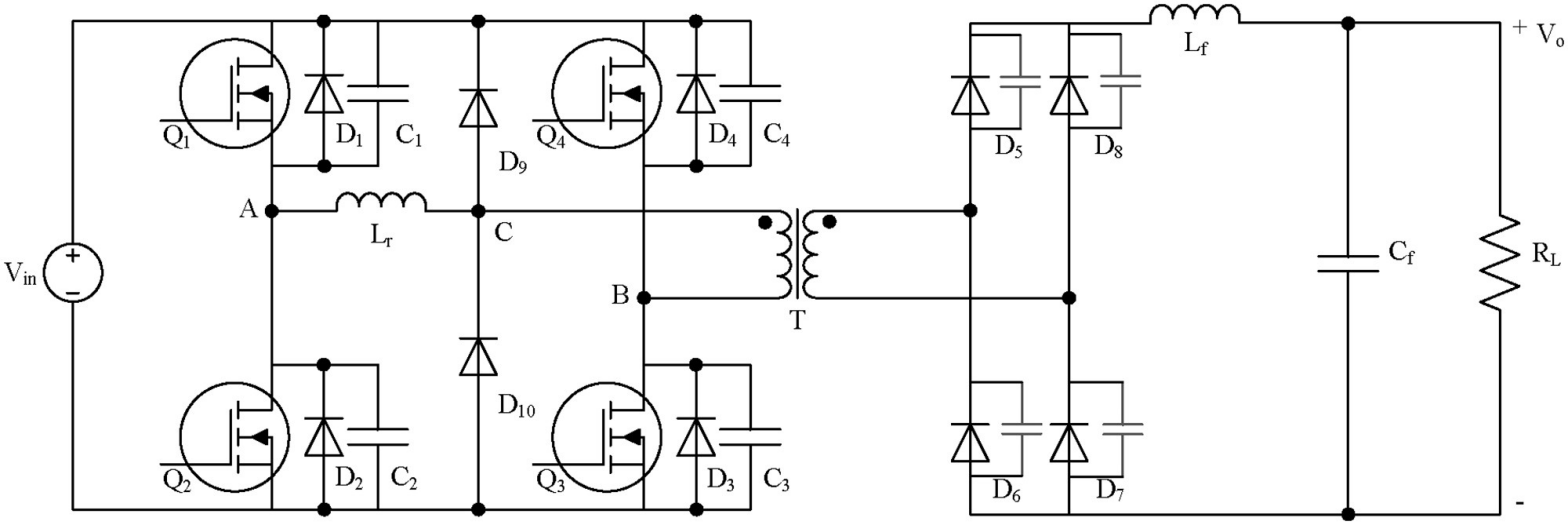
Full-Bridge DC/DC converter with primary clamping diode.

Traditional full-bridge converters can achieve soft-switching of the primary-side MOSFET, but the transformer leakage inductance resonates with the junction capacitance of the diode during circuit operation. This phenomenon leads to the output oscillation and voltage spike of the rectifier bridge, which affects the selection of the rectifier diode. Therefore, this study selected a full-bridge converter with a clamping diode in series on the primary side of the transformer [21,22], as shown in (Fig 5).

To realize a MOSFET zero-voltage switch (ZVS), there should be enough energy to meet three conditions:

1. Release the energy stored in the parasitic capacitance of the MOSFET to be turned on.
2. Charge the parasitic capacitance of the MOSFET turned off at the same leg.
3. Considering the influence of the parasitic parameters of the transformer, a part of the energy is required to release the energy stored in the distributed capacitance of the transformer winding.

Therefore, the energy provided by the inductor should meet:

$$E\;\text{>}\frac{1}{2}C_{\text{i}}V_{\text{in}}^{\text{2}}\text{+}\frac{1}{2}C_{\text{i}}V_{\text{in}}^{\text{2}}\text{+}\frac{1}{2}C_{\text{Tr}}V_{\text{in}}^{\text{2}}=C_{\text{i}}V_{\text{in}}^{\text{2}}\text{+}\frac{1}{2}C_{\text{Tr}}V_{\text{in}}^{\text{2}}$$ (16)

where *C*_i_ is the parasitic capacitance in parallel with the MOSFET(i=1,2,3,4), and *C*_Tr_ is the distributed capacitance of the transformer winding. For the leading leg, the resonant inductance L_r_ and output filter inductance L_f_ work in the equivalent series mode, and the value of L_f_ is usually large. Therefore, the two inductors together provide the energy required by the MOSFETS ZVS, which can easily meet the requirements of the above equation.

However, during the operation of the lagging leg, because the secondary rectifier diodes were all turned on, the transformer voltage was limited to 0 V, and the secondary side of the circuit was in a short-circuit state. At this time, the circuit is divided into two parts, and the output filter inductance L_f_ can no longer be converted to the primary side. The energy stored in the parasitic capacitance C_i_ and the transformer distributed capacitance C_Tr_ should be completely released by the resonant inductance L_r_. Therefore, the realization of MOSFET ZVS should satisfy the following requirements:

$$\frac{1}{2}L_{\text{r}}I_{\text{Lr}}^{\text{2}}>C_{\text{i}}V_{\text{in}}^{\text{2}}\text{+}\frac{1}{2}C_{\text{Tr}}V_{\text{in}}^{\text{2}}$$ (17)

where *I*_Lr_ is the resonant inductance current.

It can be observed that soft switching of the lagging-leg is more difficult to be achieved. On the premise of not considering the change in transformer core parameters, only a reasonable setting of the parameter of the resonant inductance L_r_ can realize the ZVS of the MOSFET on both legs.

### 3.2 Parameter determination of Full-Bridge converter with primary clamping diode

The design parameters of the full-bridge DC/DC converter are listed in Table 3.

**Table 3:** Design parameters of Full-Bridge DC/DC converter.

Design parameters	Minimum value	Typical value	Maximum value	Unit
Rated output power *P*_out_	--	100	--	W
Input voltage *V*_in_	24	24	26	V
Output voltage *V*_out_	12	14	14	V
Output voltage ripple Δ*V*_out_	-5	--	+5	%
Operating frequency *f*_s_	--	50	--	kHz
Operating maximum duty cycle *D*_s_	--	35	--	%
Target efficiency *η*	--	90	--	%

#### 3.2.1 MOSFET and rectifier diode selection

First, the maximum input current on the primary side should be calculated:

$$I_{\text{in\_max}}=\frac{P_{\text{in\_max}}}{V_{\text{in\_min}}D_{\text{max}}}$$ (18)

where *P*_in_max_ is the maximum input power, *V*_in_min_ is the minimum input voltage, and *D*_max_ is the maximum duty cycle for the entire machine operation.

Considering the target efficiency of more than 90%, *P*_in_max_= 111.11 W was calculated from the output power. Owing to the loss of duty-circle in the full-bridge converter, let *D*_max_= 2*D*_s_. Substituting it into Eq (18) we obtain *I*_in_max_= 6.613A. From this, the maximum current flowing through the MOSFET can be calculated as

$$I_{\text{mos\_rms}}=I_{\text{in\_max}}\times \sqrt{\frac{D_{\text{max}}}{\text{2}}}=3.912\;\text{A}$$ (19)

In full-bridge converters, the voltage of a single MOSFET is the primary input voltage. Based on the requirements of 1.5 times the withstand voltage and three times the overcurrent, we selected an N-channel MOSFET with an overcurrent of 18 A and a withstand voltage of 40 V, model LNL04R075.

The selection of the rectifier diode can be based on the selection method of the MOSFET. First, we calculated the RMS value of the maximum current flowing through the diode.

$$I_{\text{dio\_rms}}=I_{\text{out\_nom}}\sqrt{\frac{D_{\text{max}}}{\text{2}}}=\frac{P_{\text{out}}}{V_{\text{out}}}\sqrt{\frac{D_{\text{max}}}{\text{2}}}=4.226\;\text{A}$$ (20)

where *I*_out_nom_ is the rated output current.

Considering a margin of 1.5 times, this value is too large for a single diode; therefore, two diodes were used in parallel. Therefore, the current flowing through a single diode was 3.2 A. When the diode was cut off, the reverse voltage of the single diode was the output voltage. Based on the margin of 1.2 times, the selected rectifier diode should have at least 17V reverse withstand voltage. In summary, this study chose a fast recovery Schottky diode of type MBRD6U60CT as the rectifier diode on the secondary side.

#### 3.2.2 Selection of primary clamp diode

The clamping diode was selected according to the selection method for the rectifier diode. The reverse voltage of the clamp diode was the input voltage of the power supply. Considering a margin of 1.5 times, the clamping diode should withstand at least 36 V. Because the current flowing through the clamp diode is the difference between the resonant current and the primary current, the value is relatively small; therefore, the overcurrent requirement for the primary clamp diode can be appropriately lowered. In summary, this study chose a fast recovery diode of type ES2A as the primary clamp diode.

#### 3.2.3 High frequency transformer design

The transformer ratio should consider the situation when the input voltage is the lowest and the duty cycle is the largest.


$$N_{\text{ps}}=\frac{V_{\text{in\_min}}D_{\text{max}}}{V_{\text{out\_nom}}+V_{\text{F}}+0.5\text{V}}$$ (21)

Let *D*_max_ = 0.7, the conduction voltage drop in the rectifier diode *V*_F_ = 0.65 V, and then consider the line voltage drop of 0.5 V, then substitute into the calculation to obtain *N*_ps_ = 1.109. Here, the transformation ratio was set as 1.1.

#### 3.2.4 Design of resonant inductor

From the analysis of the full-bridge converter soft-switching implementation conditions, it is clear that the soft-switching of the MOSFETs in the lagging-leg is more difficult to achieve; therefore, the resonant inductor parameter values should be reasonably designed. Considering the distributed capacitance of the transformer and the output parasitic capacitance of the MOSFET, the equivalent parasitic capacitance *C*_r_ within a switching cycle is expressed as:

$$C_{\text{r}}\text{=2}\times C_{\text{i}}\text{+}C_{\text{Tr}}$$ (22)

By consulting the datasheet of LNL04R075 and estimation of the transformer stray capacitance, we obtained *C*_i_ = 316 pF and *C*_Tr_ = 100 pF, and by calculation we obtain *C*_r_ = 732 pF.

When the input voltage is the highest, the maximum energy stored by the equivalent parasitic capacitance is

$$E_{\text{Cr}}=\frac{1}{2}\text{×}C_{\text{r}}\text{×}V_{\text{in\_max}}^{\text{2}}=247.416\;\text{nJ}$$ (23)

Taking 2% of the switching period as the switching state transition time, the resonant inductance *L*_r_ can be determined using the following equation:

$$L_{\text{r}}=\frac{\text{2\%}\times \frac{\text{1}}{f_{\text{s}}}}{\text{2}}\times \frac{V_{\text{in\_nom}}N_{\text{ps}}}{I_{\text{out\_nom}}}=0.745\;\mu \text{H}$$ (24)

Let *L*_r_=1 μH, based on the actual situation. therein, the minimum primary current required to realize soft-switching is calculated as

$$I_{\text{ZVS}}=\sqrt{\frac{E_{\text{Cr}}}{L_{\text{r}}}}=0.497\;\text{A}$$ (25)

According to Eq (25), the current is far less than the primary current value of the circuit under normal operation; therefore, the selected resonant inductor can ensure that the MOSFET operates in the soft-switching state.

#### 3.2.5 Design of output filter inductor and filter capacitor

Because the full-bridge converter can be regarded as a derivative circuit of the Buck converter, the design of its LC filter can refer to the design method of the buck circuit, as shown in the following equation:

$$L_{\text{f}}=\frac{V_{\text{out\_nom}}}{\left(\text{2}f_{\text{s}}\right)\text{20\%}I_{\text{out\_nom}}}\left[\text{1-}\frac{V_{\text{out\_nom}}}{\frac{V_{\text{in\_max}}}{N_{\text{ps}}}\text{-}V_{\text{D}}\text{-}V_{\text{Lf}}}\right]$$ (26)

where *V*_out_nom_ is the rated output voltage, *f*_s_ the switching frequency, *V*_in_max_ the maximum input voltage, *N*_ps_ the transformer ratio, *V*_D_ the diode voltage drop, and *V*_Lf_ the filtered inductive voltage drop. Considering actual conditions, *L*_f_ =50 μH.

The design of the output filter capacitor mainly should consider the output voltage ripple requirements, as analyzed based on the following equation:

$$C_{\text{f}}=\frac{V_{\text{out\_min}}}{\text{8}L_{\text{f}}{\left(\text{2}f_{\text{s}}\right)}^{\text{2}}\Delta V}\left[\text{1-}\frac{V_{\text{out\_min}}}{\frac{V_{\text{in\_max}}}{N_{\text{ps}}}\text{-}V_{\text{D}}\text{-}V_{\text{Lf}}}\right]$$ (27)

where Δ*V* is the output voltage ripple.

Let Δ*V* = 5%, *V*_out_nom_ = 0.7V, and calculate *C*_f_ = 2 μF. To eliminate the high-frequency component of the output voltage, it is necessary to ensure that the turning frequency of the output filter is less than 10 times the switching frequency.


$$\frac{\text{1}}{\text{2π}\sqrt{L_{\text{f}}C_{\text{f}}}}\text{<}\frac{f_{\text{s}}}{\text{10}}$$ (28)

In summary, *C*_f_ = 100μF.

## 4 Control strategy of two-stage AC/DC converter

### 4.1 Control strategy design

**Fig 6:**
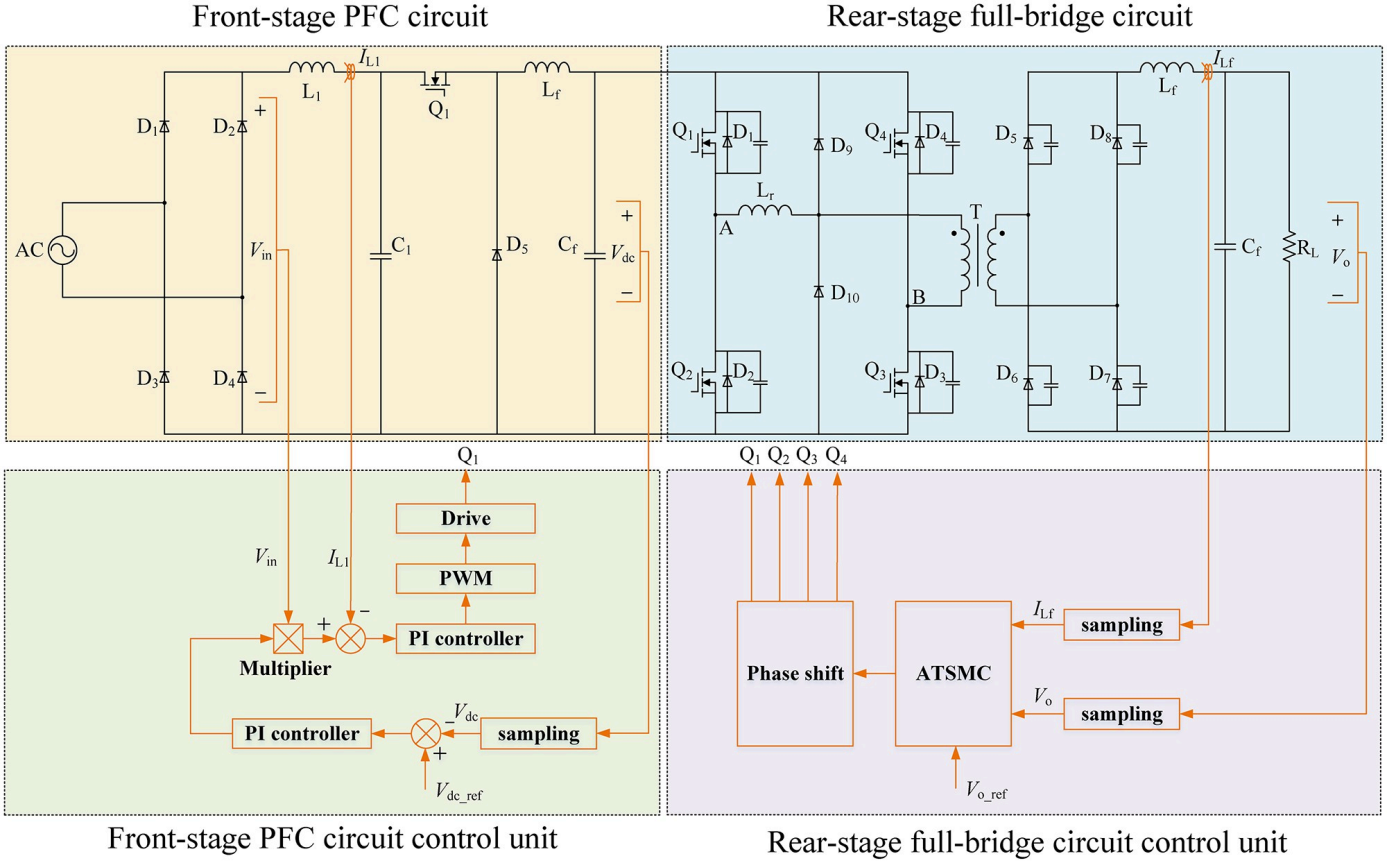
Control strategy of two-stage AC/DC converter.

The two-stage AC/DC converter control strategy is illustrated in (Fig 6).

The front-stage PFC circuit adopts a PI double-closed-loop control strategy [23]. The output voltage, input voltage, and input current were collected as the control variables. The given value of the outer loop voltage was obtained by comparing the output voltage *V*_dc_ with the reference voltage *V*_dc_ref_ in real time. After calculation by the voltage PI controller, it was multiplied by the input voltage *V*_in_ to obtain the current reference whose phase was consistent with the input voltage. Then, the current reference was compared with the input current *I*_L_ as the given value of the current inner loop. After calculation by the current PI controller, it was output to the PWM generator to generate the driving waveform to control Q_1_. This strategy realized the tracking of the current phase and the stabilization of the output voltage.

**Fig 7:**
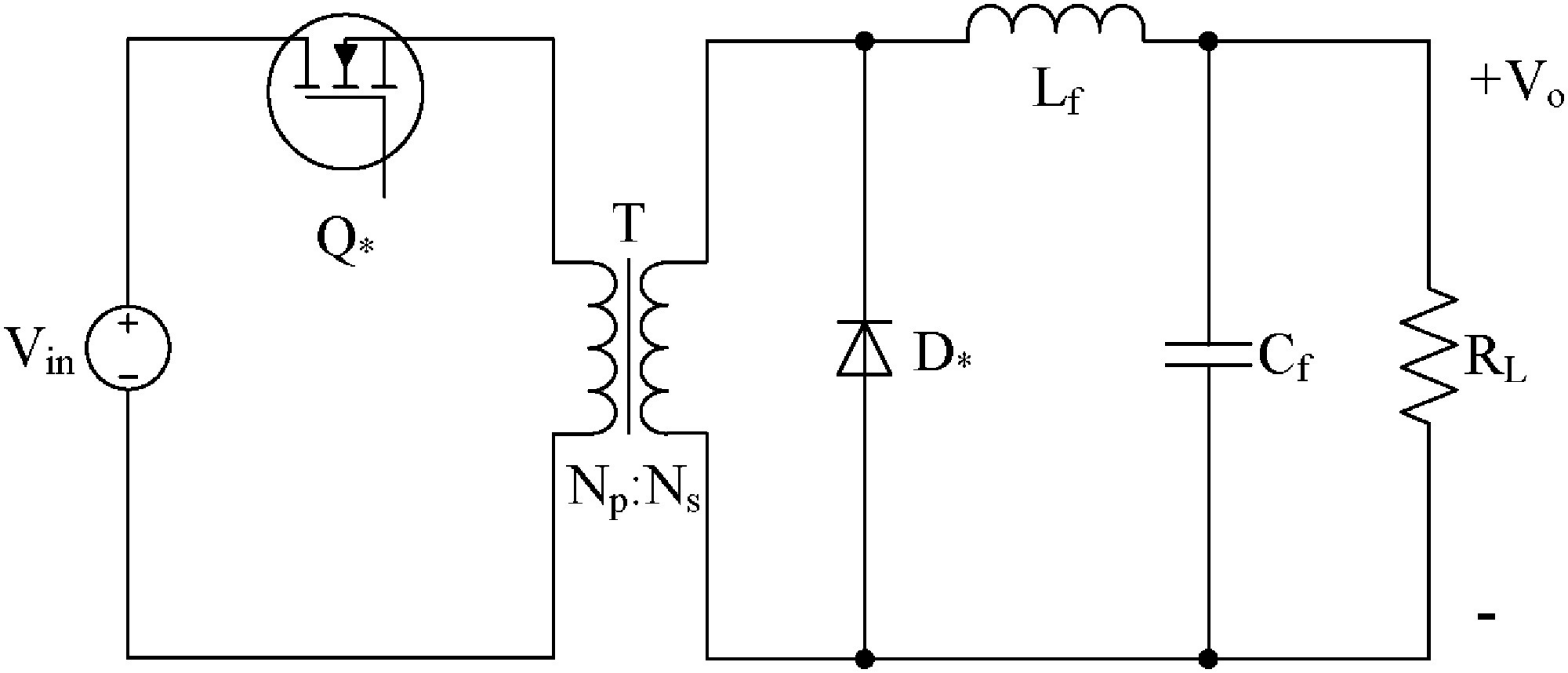
Buck circuit equivalent model of full-bridge converter.

The rear-stage full-bridge circuit adopts a parameter adaptive terminal sliding mode control (ATSMC) strategy [24–26]. The full-bridge converter was derived from the Buck circuit, so the equivalent circuit topology is shown in (Fig 7).

Q* represents the equivalent MOSFET of the full-bridge converter. Based on the state-space averaging method, the equation describing the working state of the circuit can be obtained as follows:

$$\frac{di_{\text{L}}}{dt}=\frac{V_{\text{in}}}{N_{\text{ps}}L_{\text{f}}}u-\frac{V_{\text{o}}}{L_{\text{f}}}$$ (29)

$$\frac{dV_{\text{o}}}{dt}=\frac{i_{\text{Lf}}}{C_{\text{f}}}-\frac{V_{\text{o}}}{R_{\text{L}}C_{\text{f}}}$$ (30)

where *u* is the equivalent switching control law of switch Q *. When *u* = 1, switch Q * is on, and when *u* = 0, it is OFF. *N*_ps_ represents the transformer ratio, *N*_ps_=*N*_p_/*N*_s_.

The output voltage error *x*_1_ and its rate of change *x*_2_ are defined as follows:

$$x_{1}=V_{\text{o}}-V_{\text{ref}}$$ (31)

$$x_{2}={\dot{x}}_{1}={\dot{V}}_{\text{o}}-{\dot{V}}_{\text{ref}}\approx {\dot{V}}_{\text{o}}$$ (32)

where *V*_ref_ is the reference value of the output voltage, ${\dot{x}}_{1}$ represents the derivative of *x*_1_ to time.

Subsequently, the equivalent mathematical model of the full-bridge converter is expressed as

$$\left[\begin{array}{c} {\dot{x}}_{1}\\ {\dot{x}}_{2} \end{array}\right]=\left[\begin{array}{cc} 0 & 1\\ -\frac{1}{L_{\text{f}}C_{\text{f}}} & -\frac{1}{R_{\text{L}}C_{\text{f}}} \end{array}\right]\left[\begin{array}{c} x_{1}\\ x_{2} \end{array}\right]+\left[\begin{array}{c} 0\\ \frac{uV_{\text{i}}-N_{\text{ps}}V_{\text{ref}}}{N_{\text{ps}}L_{\text{f}}C_{\text{f}}} \end{array}\right]$$ (33)

To make the system state tracking error approach zero in a finite time, a nonlinear function was introduced in the design of the sliding surface. Sliding surface function *S* is defined as follows:

$$S=\overset{\cdot }{x_{\text{1}}^{\gamma }}\text{+}k_{\text{a}}x_{\text{1}}^{\gamma }\text{+}k_{\text{b}}\overset{\text{t}}{\underset{0}{\int }}x_{\text{1}}^{\gamma }\text{d}\tau$$ (34)

$$k_{\text{a}}<0,k_{\text{b}}<0,0<\gamma <1$$ (35)

where *K*_a_ and *K*_b_ are sliding mode coefficients.

According to the Lyapunov stability criterion, to ensure that the system state trajectory can reach and remain on the sliding surface, the system state should satisfy the following requirements:

$$S\dot{S}<0$$ (36)

Here, the Lyapunov function was selected as $\text{V}=\frac{1}{2}S^{2}$, then $\dot{V}=S\dot{S}$. Taking the derivative of Eq (34) and substituting Eq (33) into it, we can get:

$$\dot{S}=\frac{\gamma V_{\text{in}}}{N_{\text{ps}}L_{\text{f}}C_{\text{f}}^{\gamma }}{\left(i_{\text{Lf}}-\frac{V_{\text{o}}}{R_{\text{L}}}\right)}^{\gamma -1}u-\frac{\gamma V_{\text{o}}}{L_{\text{f}}C_{\text{f}}^{\gamma }}{\left(i_{\text{Lf}}-\frac{V_{\text{o}}}{R_{\text{L}}}\right)}^{\gamma -1}-\frac{\gamma }{R_{\text{L}}C_{\text{f}}^{\gamma +1}}{\left(i_{\text{Lf}}-\frac{V_{\text{o}}}{R_{\text{L}}}\right)}^{\gamma }+k_{a}\gamma x_{1}^{\gamma -1}\frac{1}{C_{\text{f}}}\left(i_{\text{Lf}}-\frac{V_{\text{o}}}{R_{\text{L}}}\right)+k_{b}x_{1}^{\gamma }$$ (37)

When *u*=1, *S*<0, need $\dot{S}>0$

$$\dot{S}=\frac{\left(V_{\text{in}}-N_{\text{ps}}V_{\text{o}}\right)\gamma }{N_{\text{ps}}L_{\text{f}}C_{\text{f}}^{\gamma }}{\left(i_{\text{Lf}}-\frac{V_{\text{o}}}{R_{\text{L}}}\right)}^{\gamma -1}-\frac{\gamma }{R_{\text{L}}C_{\text{f}}^{\gamma +1}}{\left(i_{\text{Lf}}-\frac{V_{\text{o}}}{R_{\text{L}}}\right)}^{\gamma }+k_{a}\gamma x_{1}^{\gamma -1}\frac{1}{C_{\text{f}}}\left(i_{\text{Lf}}-\frac{V_{\text{o}}}{R_{\text{L}}}\right)+k_{b}x_{1}^{\gamma }>0$$ (38)

When *u*=0, *S*>0, need $\dot{S}<0$

$$\dot{S}=-\frac{\gamma V_{\text{o}}}{L_{\text{f}}C_{\text{f}}{}^{\gamma }}{\left(i_{\text{Lf}}-\frac{V_{\text{o}}}{R_{\text{L}}}\right)}^{\gamma -1}-\frac{\gamma }{R_{\text{L}}C_{\text{f}}^{\gamma +1}}{\left(i_{\text{Lf}}-\frac{V_{\text{o}}}{R_{\text{L}}}\right)}^{\gamma }+k_{a}\gamma x_{1}^{\gamma -1}\frac{1}{C_{\text{f}}}\left(i_{\text{Lf}}-\frac{V_{\text{o}}}{R_{\text{L}}}\right)+k_{b}x_{1}^{\gamma }<0$$ (39)

If the values of *k*_a_ and *k*_b_ satisfy the above inequality equations, the Lyapunov stability condition can be satisfied. Let *γ* ≈ 1, *i*_*Lf*_ ≈ 0, the value range of *k*_a_ and *k*_b_ can be deduced as

$$\frac{1}{R_{\text{L}}C_{\text{f}}}-\frac{R_{\text{L}}}{L_{\text{f}}}<k_{\text{a}}<0$$ (40)

$$\frac{{\left(R_{\text{L}}C_{\text{f}}\right)}^{2}-1+R_{\text{L}}C_{\text{f}}k_{\text{a}}}{{\left(R_{\text{L}}C_{\text{f}}\right)}^{3}}<k_{\text{b}}<0$$ (41)

When *S*=0, the system is in sliding mode. Let $\dot{S}=0$, and the equivalent control law *u*_eq_ can be deduced from Eq (37).


$$u_{eq}=\frac{N_{\text{ps}}}{V_{\text{in}}}\left[V_{\text{o}}+\frac{L_{\text{f}}}{R_{\text{L}}C_{\text{f}}}\left(i_{\text{Lf}}-\frac{V_{\text{o}}}{R_{\text{L}}}\right)-k_{\text{a}}L_{\text{f}}C_{\text{f}}^{\gamma \text{-1}}x_{1}^{\gamma \text{-1}}{\left(i_{\text{Lf}}-\frac{V_{\text{o}}}{R_{\text{L}}}\right)}^{2-\gamma }-\frac{L_{\text{f}}k_{\text{b}}}{\gamma }{\left(C_{\text{f}}x_{1}\right)}^{\gamma }{\left(i_{\text{Lf}}-\frac{V_{\text{o}}}{R_{\text{L}}}\right)}^{1-\gamma }\right]$$ (42)

In summary, the system control law can be written as

$$u=\left\{\begin{array}{cc} 1 & S<0\\ u_{eq} & S=0\\ 0 & S>0 \end{array}\right.$$ (43)

In addition, different values of γ have a significant influence on the system performance. When the value of γ increases, the response speed of the system increases, however this also leads to a large voltage overshoot. Conversely, when the value of γ decreases, the overshoot decreases in the system startup phase; however, the voltage drop increases when a disturbance occurs. This is in contrast to the desired control effect. It can be observed that a fixed γ value cannot guarantee the optimal control effect of the system. Therefore, an improved adaptive algorithm for the γ parameter is proposed, as presented in Eq (44).


$$\gamma =\frac{1}{\pi }\text{arctan}\left(\lambda x_{1}-1\right)+0.5\;\;\;\;\left(\lambda >0\right)$$ (44)

To improve the rapidity of the system, the λ factor was introduced to improve the approach speed of the γ-adaptive algorithm in different situations to adjust the dynamic performance of the system and achieve stable voltage output.

### 4.2 Simulation of front-stage PFC circuit

**Fig 8:**
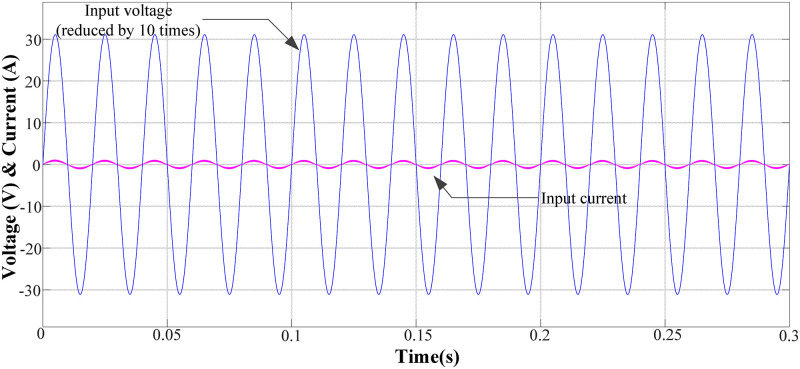
Input voltage and current waveforms in normal operation mode.

**Fig 9:**
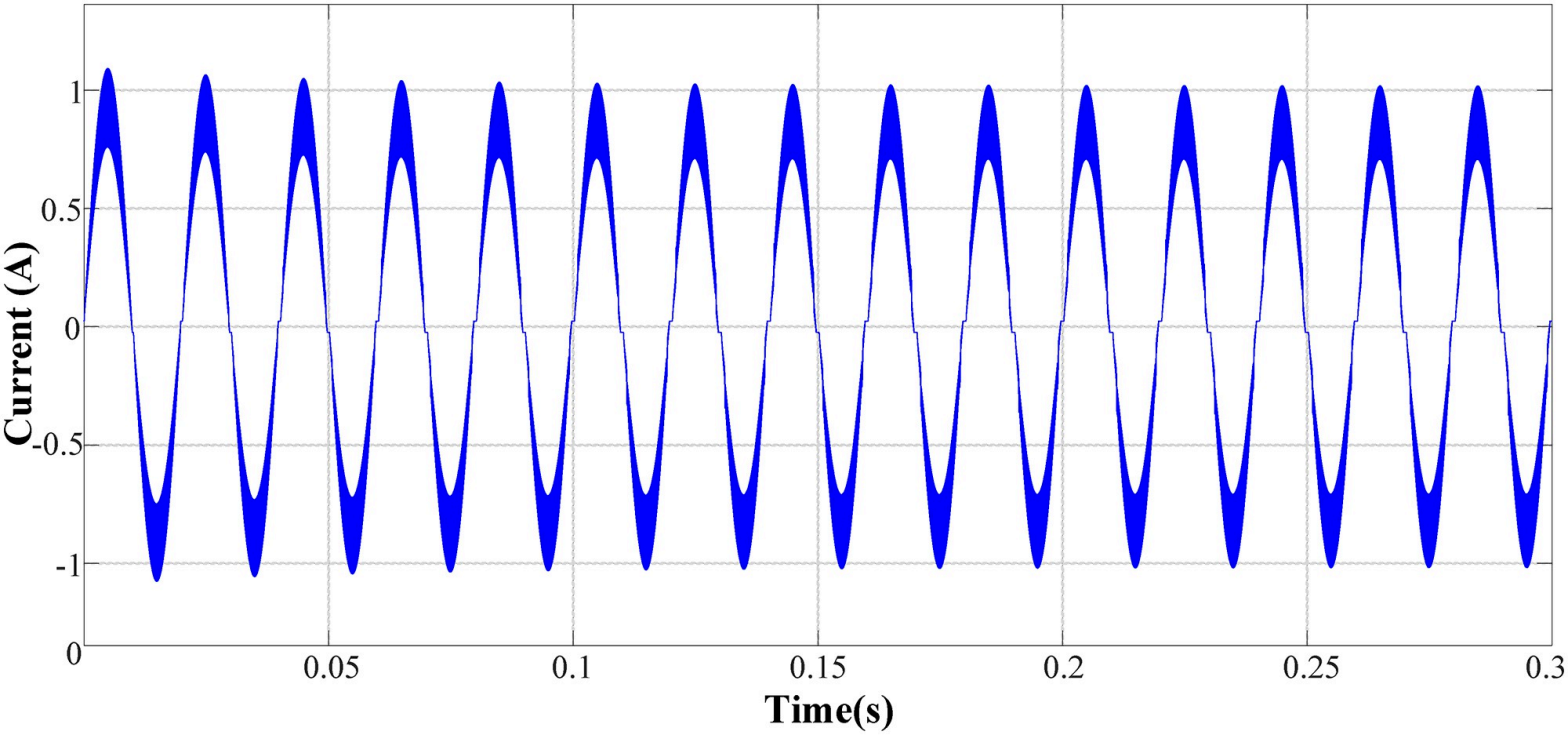
Input current waveform in normal operation mode.

The system is simulated based on MATLAB. By engineering turning method, the voltage outer-loop coefficients were determined as *K*_p_=0.1 and *K*_i_=0.5, and the current inner-loop coefficients were *K*_p_=0.08 and *K*_i_=4.

(Fig 8) shows the input voltage and current waveform of the circuit, where the peak value of the input voltage was 311 V. At this time, the power factor reached 99.03%, which meets the design requirements.

(Fig 9) shows the input current waveform. From the figure, the input current still has a certain degree of waveform distortion at the switching time of the positive and negative half cycles. The total harmonic distortion of the current is 16.46%, but each harmonic distortion meets the IEC61000-3-2 Class C standard, meeting the design requirements.

**Fig 10:**
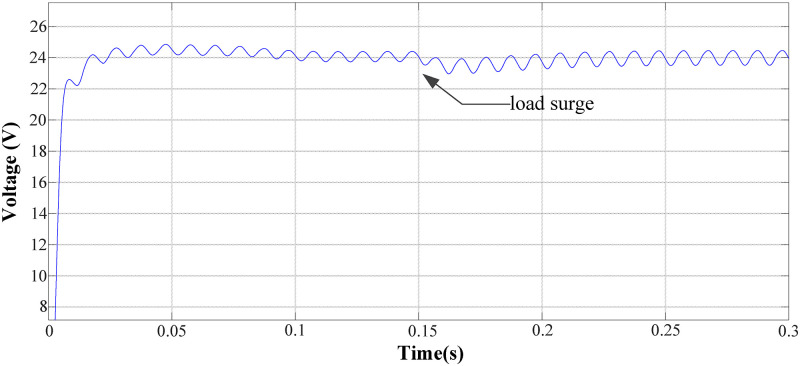
System output voltage waveform under load surge.

To verify the system recovery effect under a load disturbance, two typical conditions of load surge and load drop were simulated. The output voltage waveform of the circuit when the load surges is shown in (Fig 10). Through quantitative analysis, the output voltage can be adjusted within 100 ms, regardless of the load surge or drop. The absolute value of the maximum voltage fluctuation does not exceed 1.2V, and the output voltage ripple at the steady state is less than 5%. Therefore, the design requirements were met.

### 4.3 Simulation of rear-stage full-bridge converter

**Fig 11:**
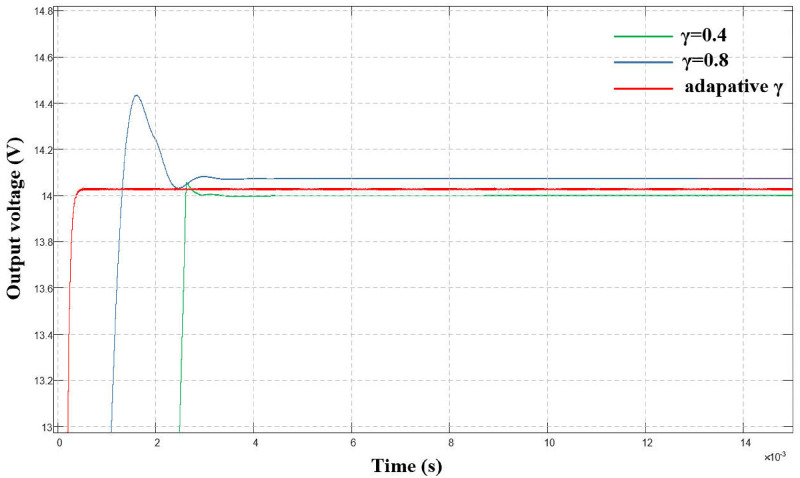
Comparison of output voltage waveforms at system startup.

**Fig 12:**
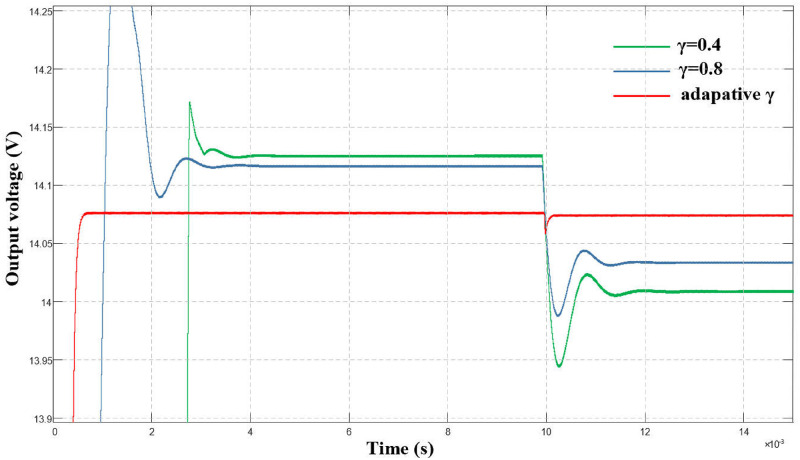
Comparison of output voltage waveform under load disturbance.

Based on the previous analysis, considering the stability and rapidity of the system, *λ* = 4, *k*_a_ = -4 × 10^4^, and *k*_b_ = -3 × 10^10^ were selected.

(Fig 11) shows a comparison of the output voltage waveform between γ with a fixed value and the adaptive algorithm when the system is started. Clearly from the figure, compared to using a fixed γ value, the output can better track the system state using an adaptive algorithm to obtain γ value, which improves the response speed while considering system stability.

(Fig 12) shows a comparison of the output voltage waveform with a fixed γ value and the adaptive algorithm when the load generates a disturbance. The load disturbance was observed at 0.01 s. Under the adaptive algorithm, the system output voltage drop was smaller, the disturbance recovery time was shorter, and the system robustness was significantly enhanced.

## 5 Experimental verification

**Fig 13:**
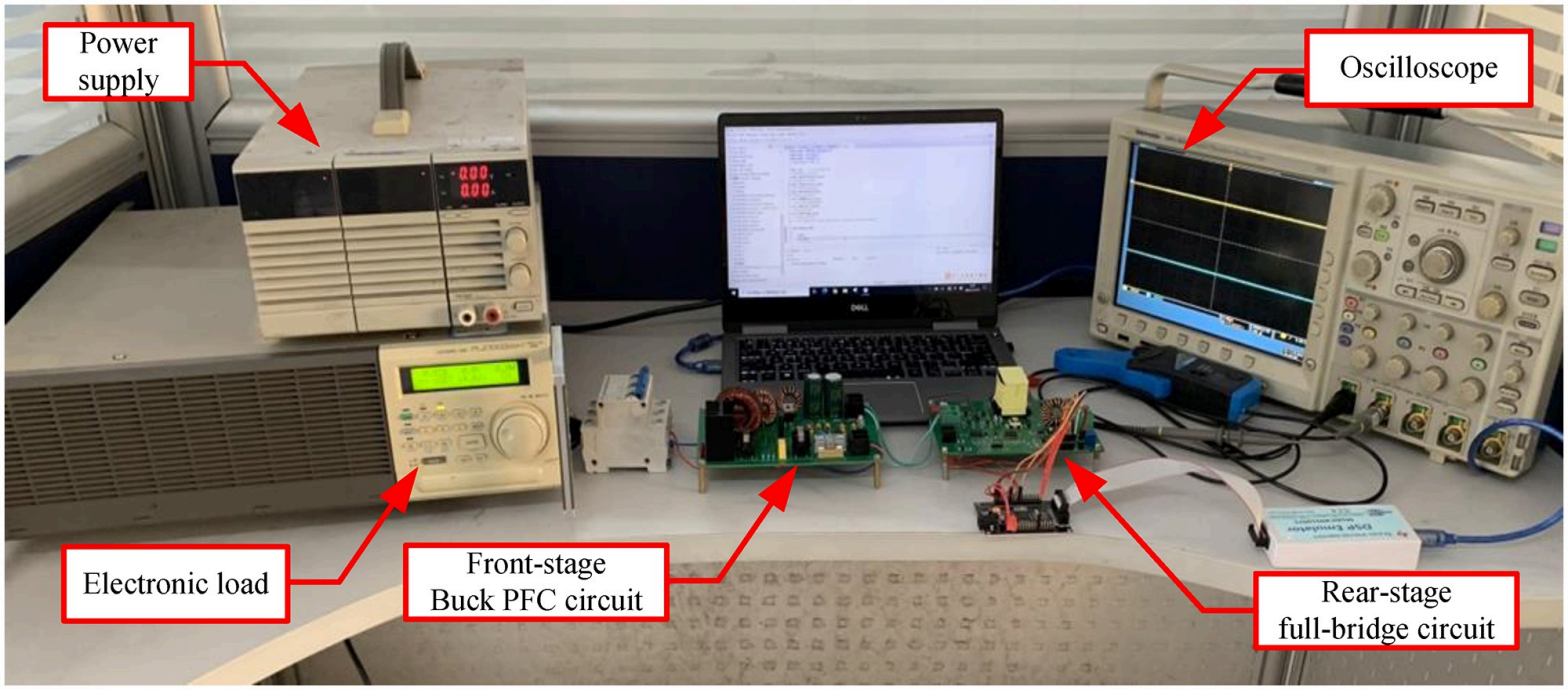
Prototype platform of two-stage AC/DC converter.

Based on the previous analysis, an experimental prototype of a two-stage isolated AC/DC converter was built. The experimental setup is illustrated in (Fig 13).

**Fig 14:**
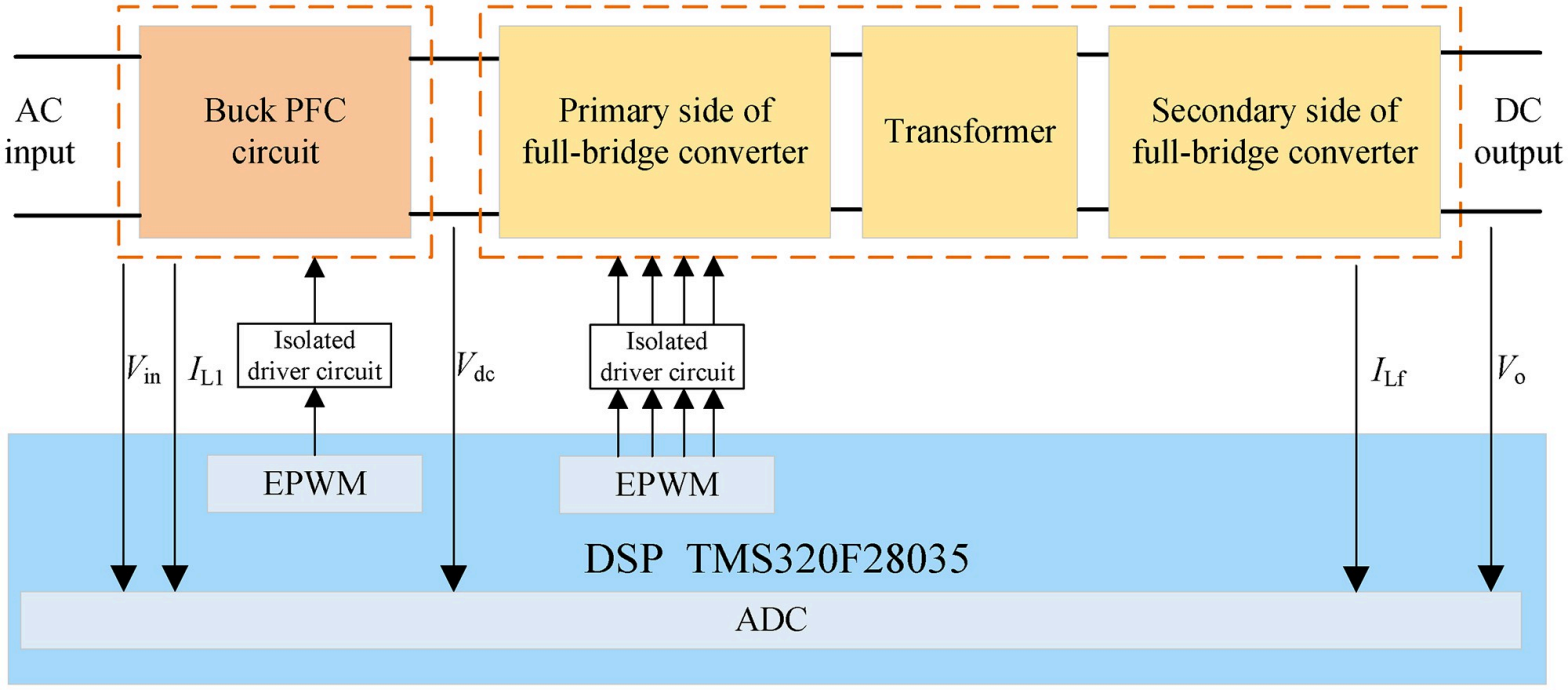
Software and hardware control flow chart.

The control strategy is realized digitally by DSP. The control flow is shown in (Fig 14). TMS320F28035 is used as the main chip because of its powerful performance and its accompanying ADC module and EPWM module. The sampled voltage and current signals first enter the ADC module for processing. After the DSP calculation, the PWM drive signal is output to the isolation drive circuit through the EPWM module, and finally the control of the MOSFET is realized.

**Fig 15:**
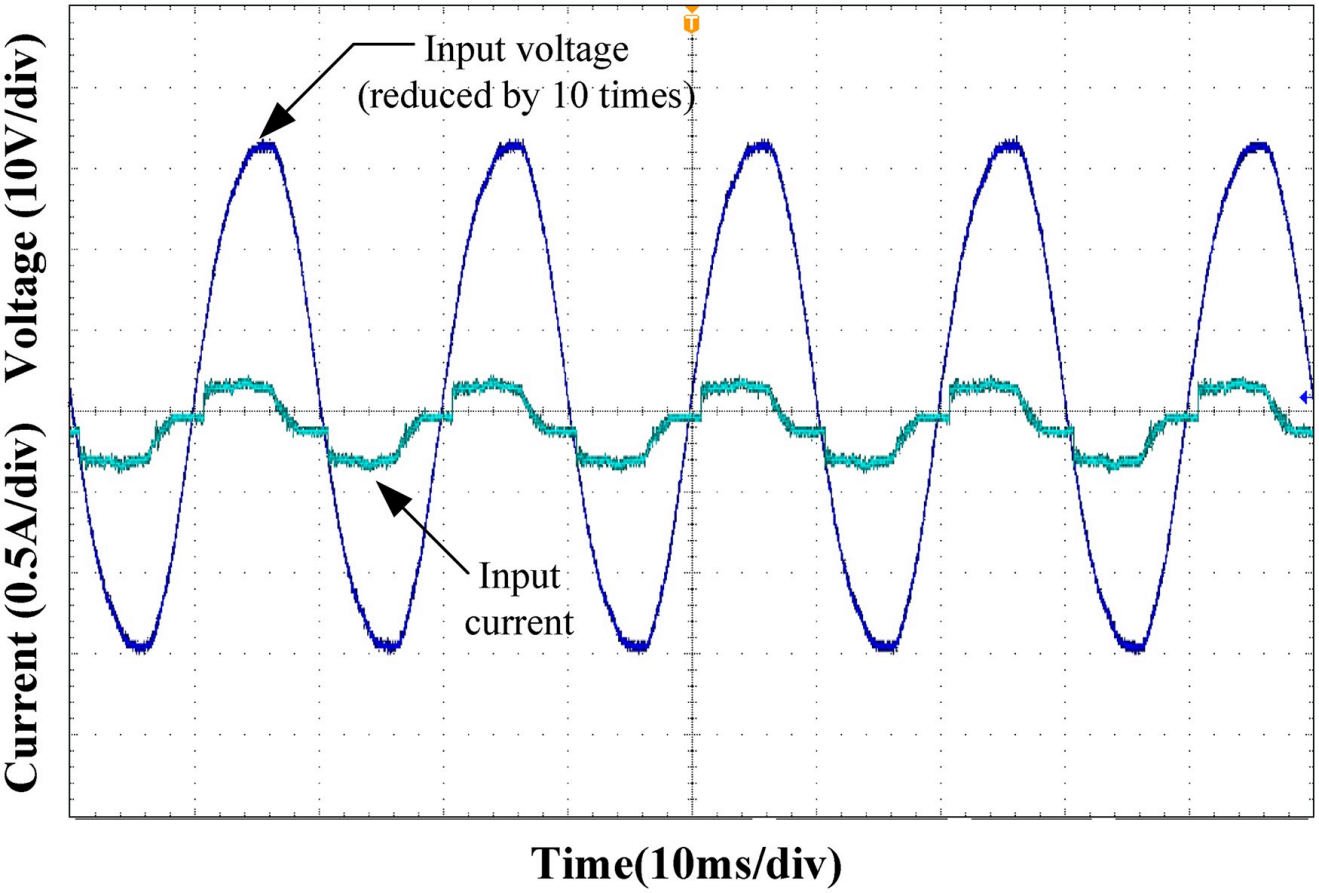
Input voltage and current at 25% load.

**Fig 16:**
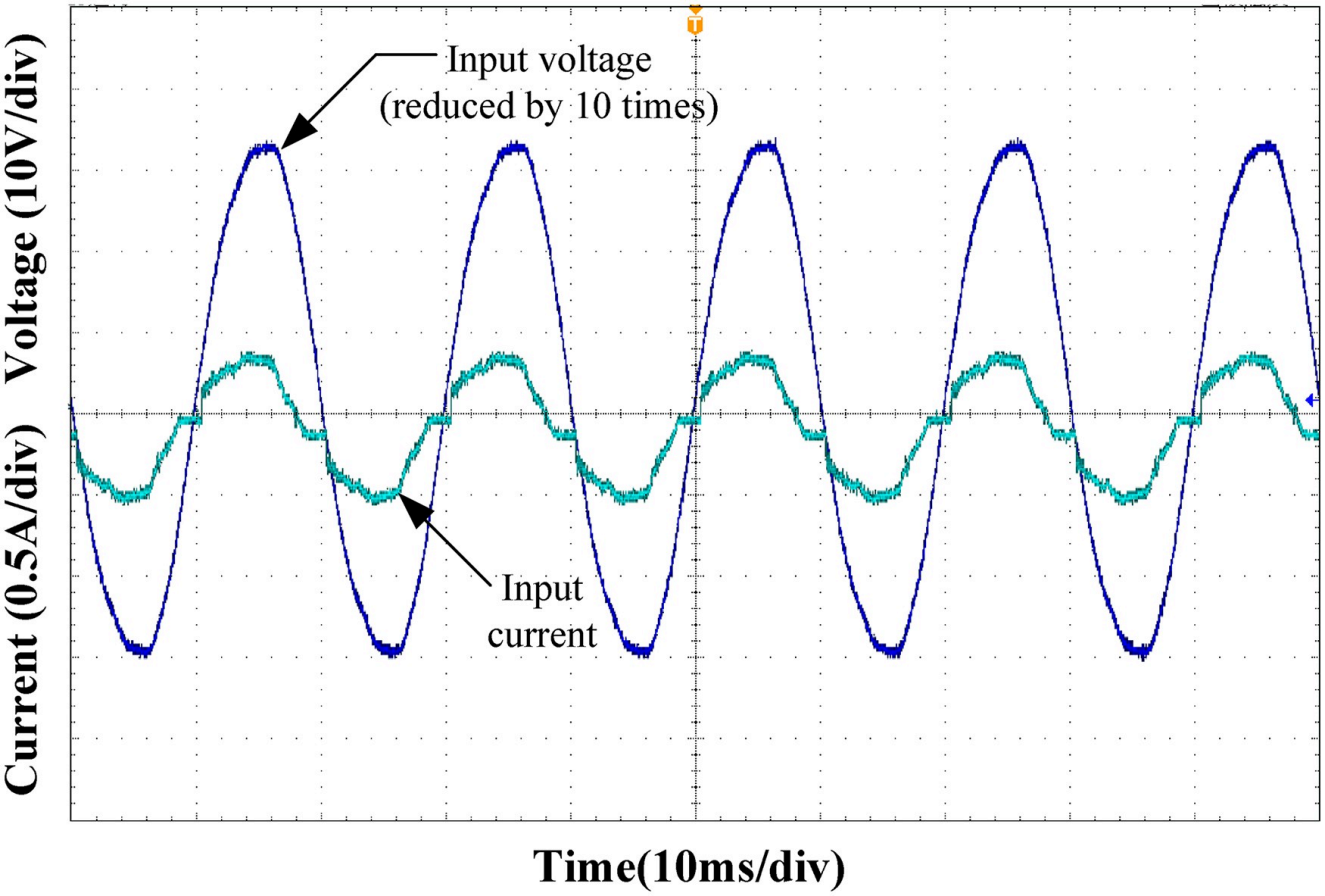
Input voltage and current at 50% load.

**Fig 17:**
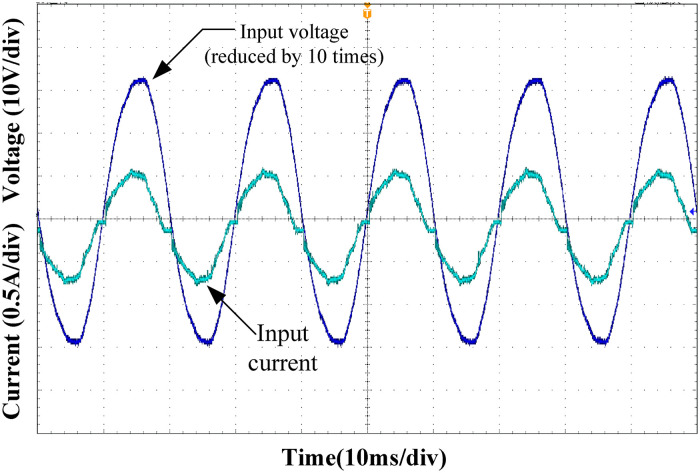
Input voltage and current at 75% load.

**Fig 18:**
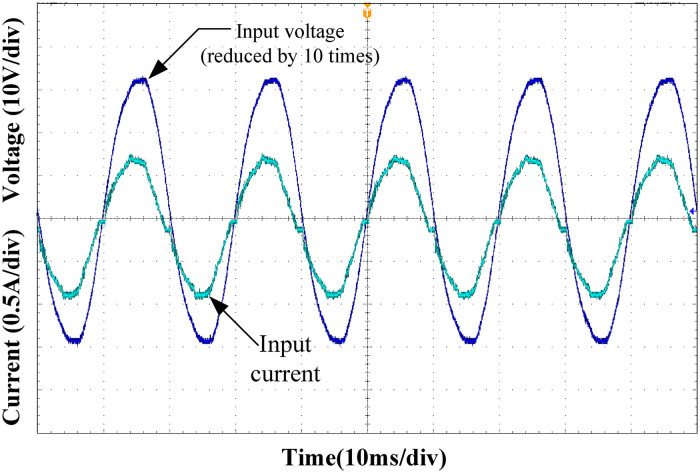
Input voltage and current at full load.

Figs 15–18 show the input voltage and current waveforms under different load conditions. Clearly from the figure, the designed Buck PFC circuit can achieve power factor correction under different operating conditions. As the load power increased, the input current THD decreased, and the power factor of the system increased, satisfying the input requirements of the rear-stage DC/DC converter.

**Fig 19:**
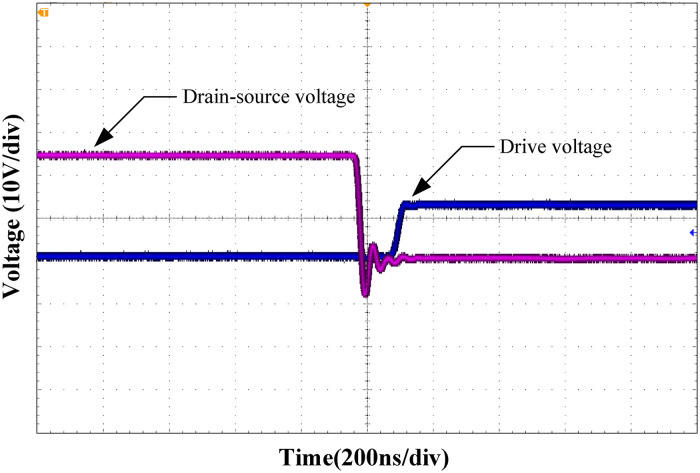
Zero-voltage turn-on waveform of the leading-leg.

**Fig 20:**
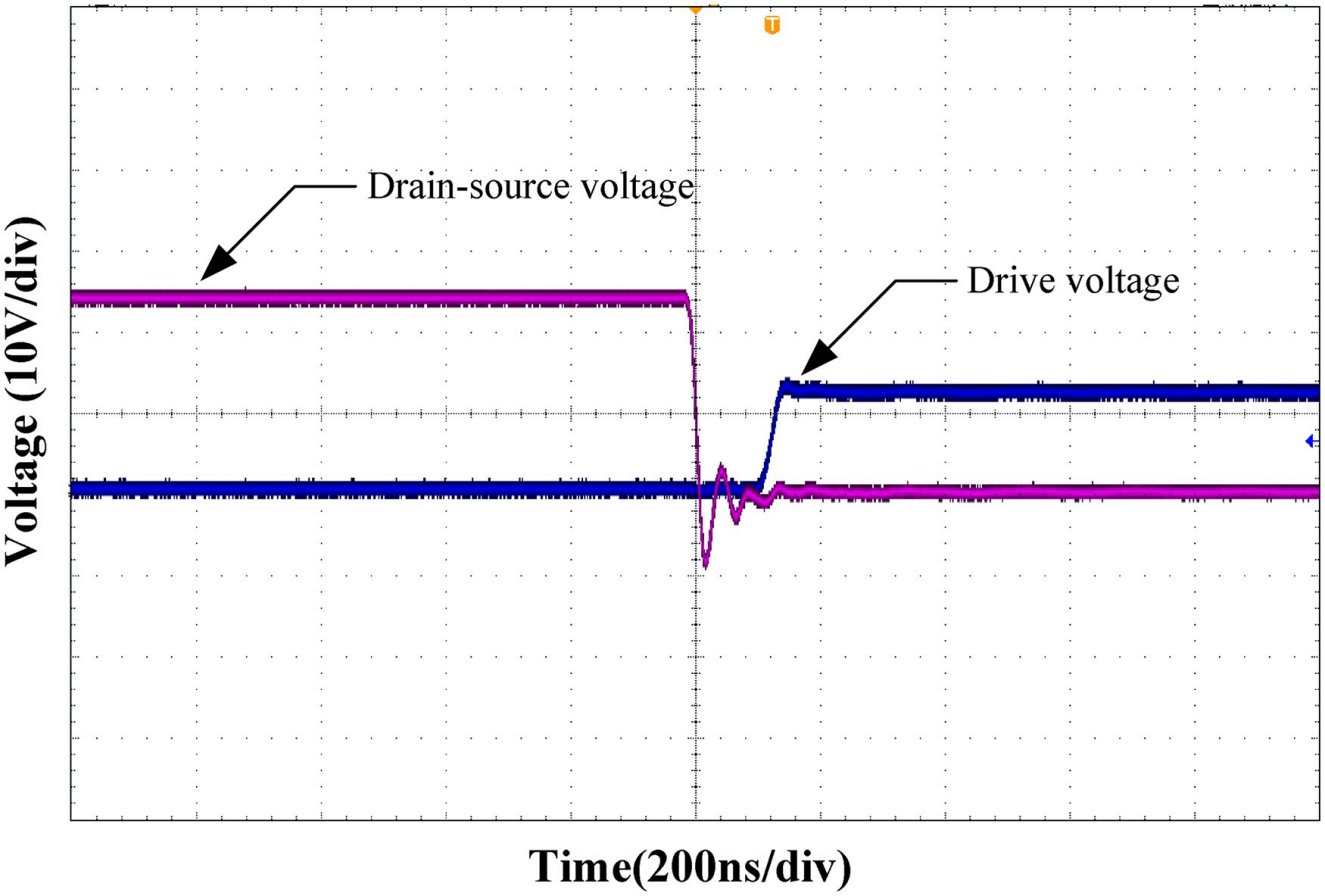
Zero-voltage turn-on waveform of the lagging-leg.

**Fig 21:**
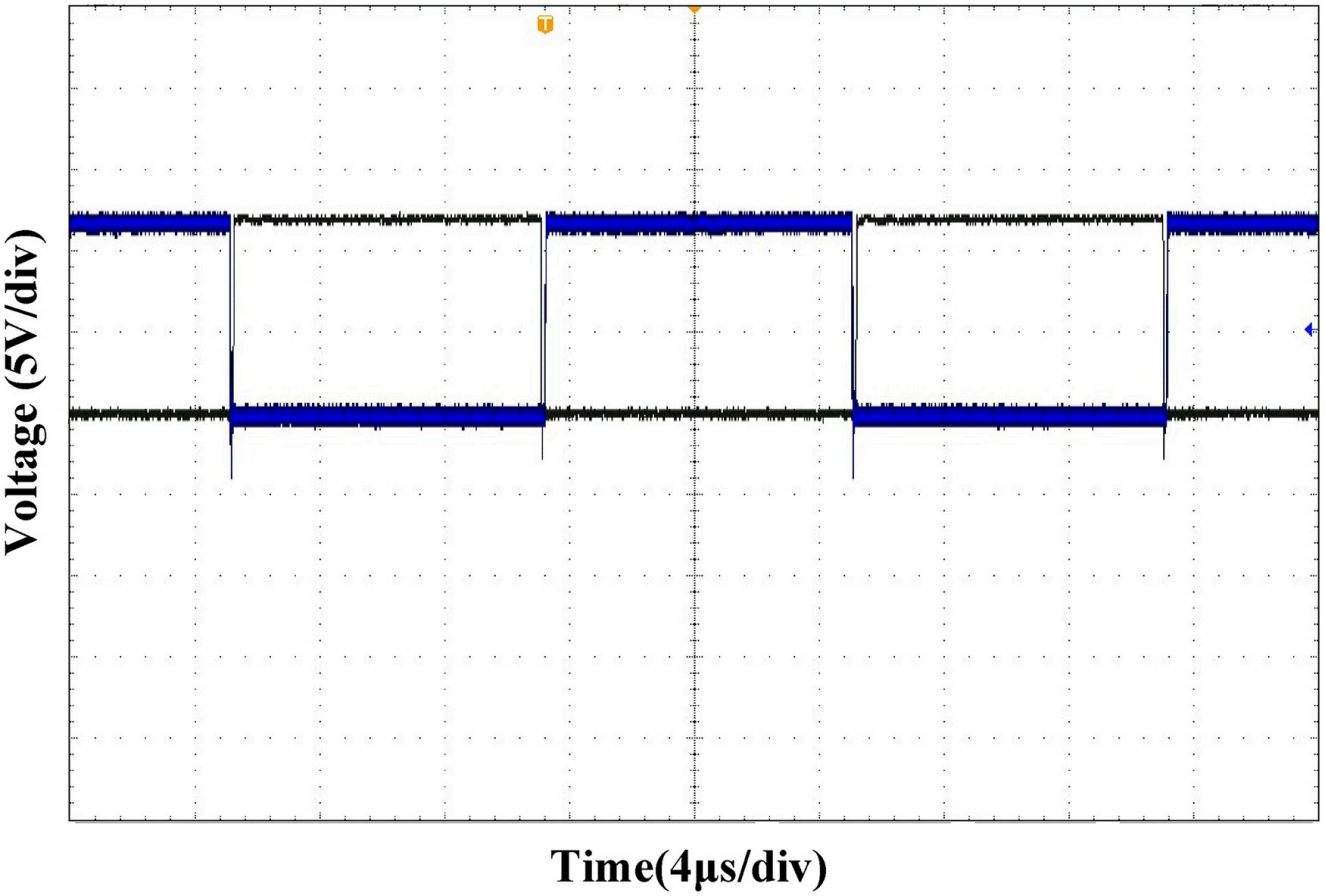
MOSFET driving waveform of the leading-leg.

**Fig 22:**
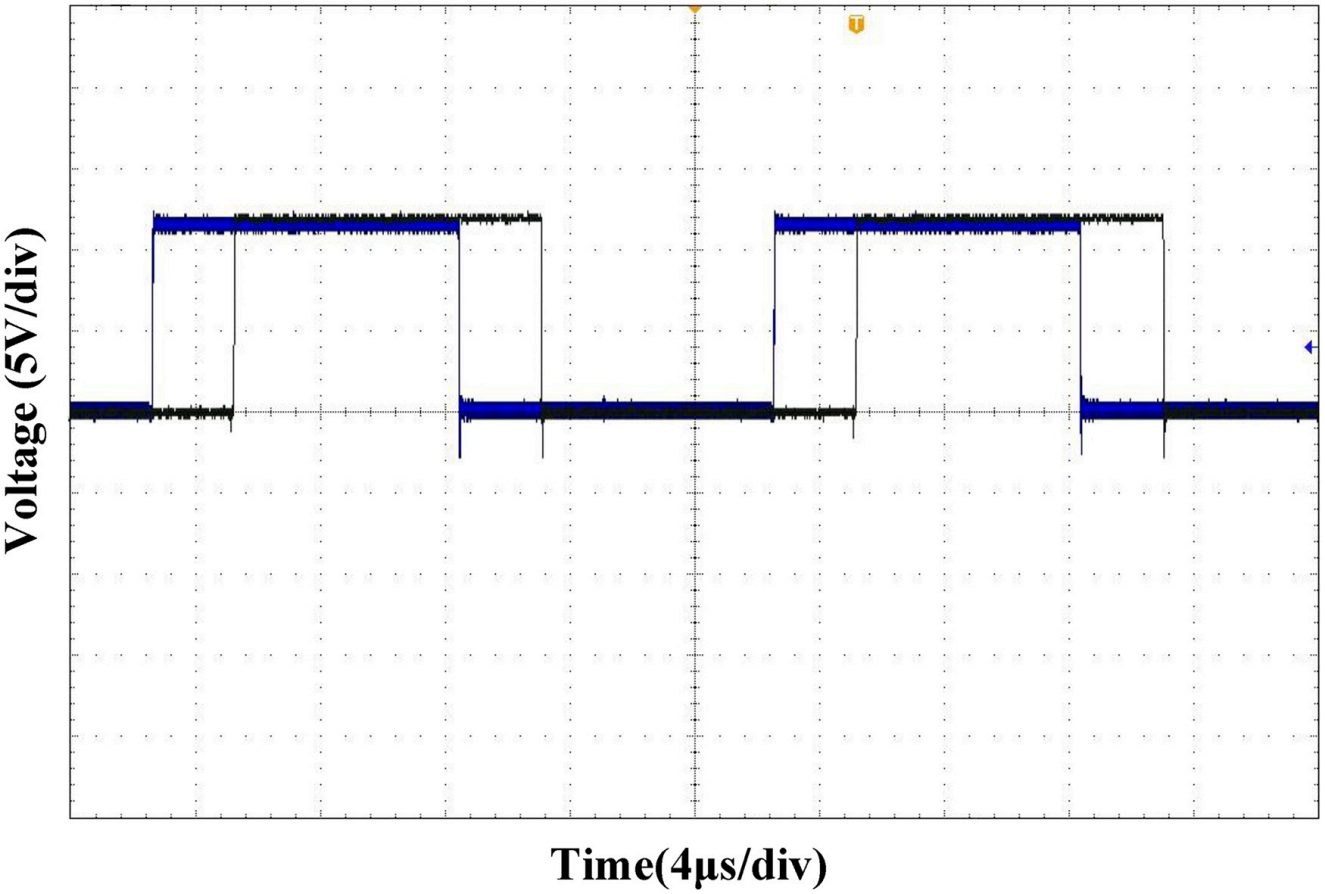
MOSFET driving waveforms of the leading-leg and lagging-leg.

Figs 19 and 20 show the zero-voltage turn-on waveforms of the leading and lagging legs, respectively. As shown from the figure, when the control signal of the MOSFET arrives, the drain-source voltage drops to zero, realizing the ZVS of the MOSFET.

(Fig 21) shows the driving waveforms of MOSFET Q_1_ and Q_2_ in the leading-leg. By introducing dead-time, the shoot-through of the MOSFET is avoided, and sufficient time is reserved for the ZVS. (Fig 22) shows the driving waveforms of MOSFET Q_1_ and Q_3_ of the leading and lagging legs. It can be observed that the driving waveform has a certain phase difference, based on which the output voltage can be adjusted.

**Fig 23:**
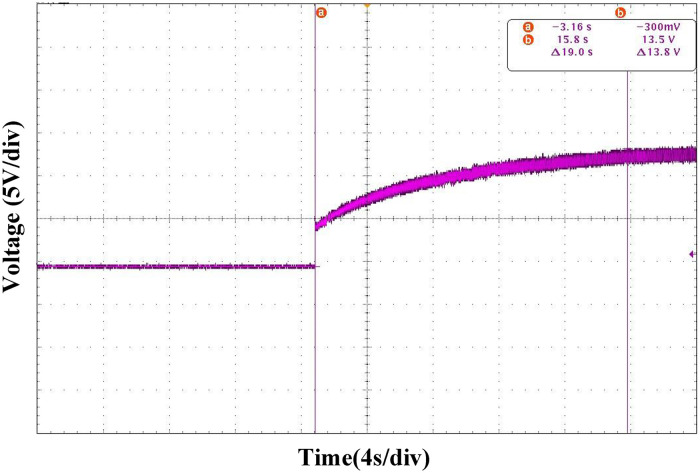
Startup waveform under PI control strategy.

**Fig 24:**
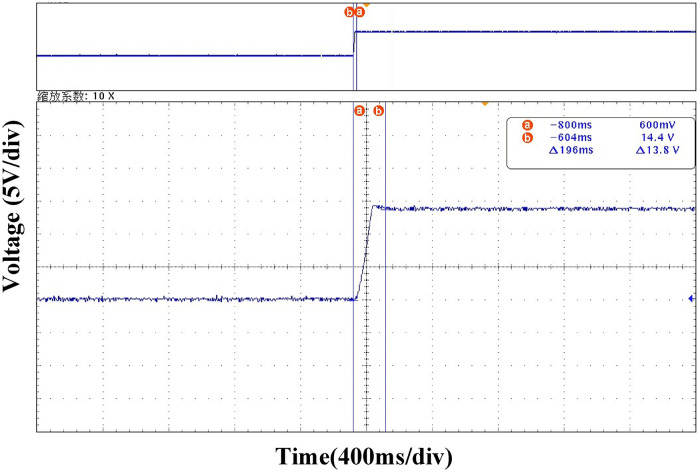
Startup waveform under ATSMC control strategy.

Figs 23 and 24 show the comparison of the converter startup waveforms under the PI and ATSMC control strategies, respectively. Using the new sliding-mode control strategy, it only takes 196ms to reach the steady state. Therefore, the new control strategy has a faster response speed and better dynamic performance during the startup phase.

**Fig 25:**
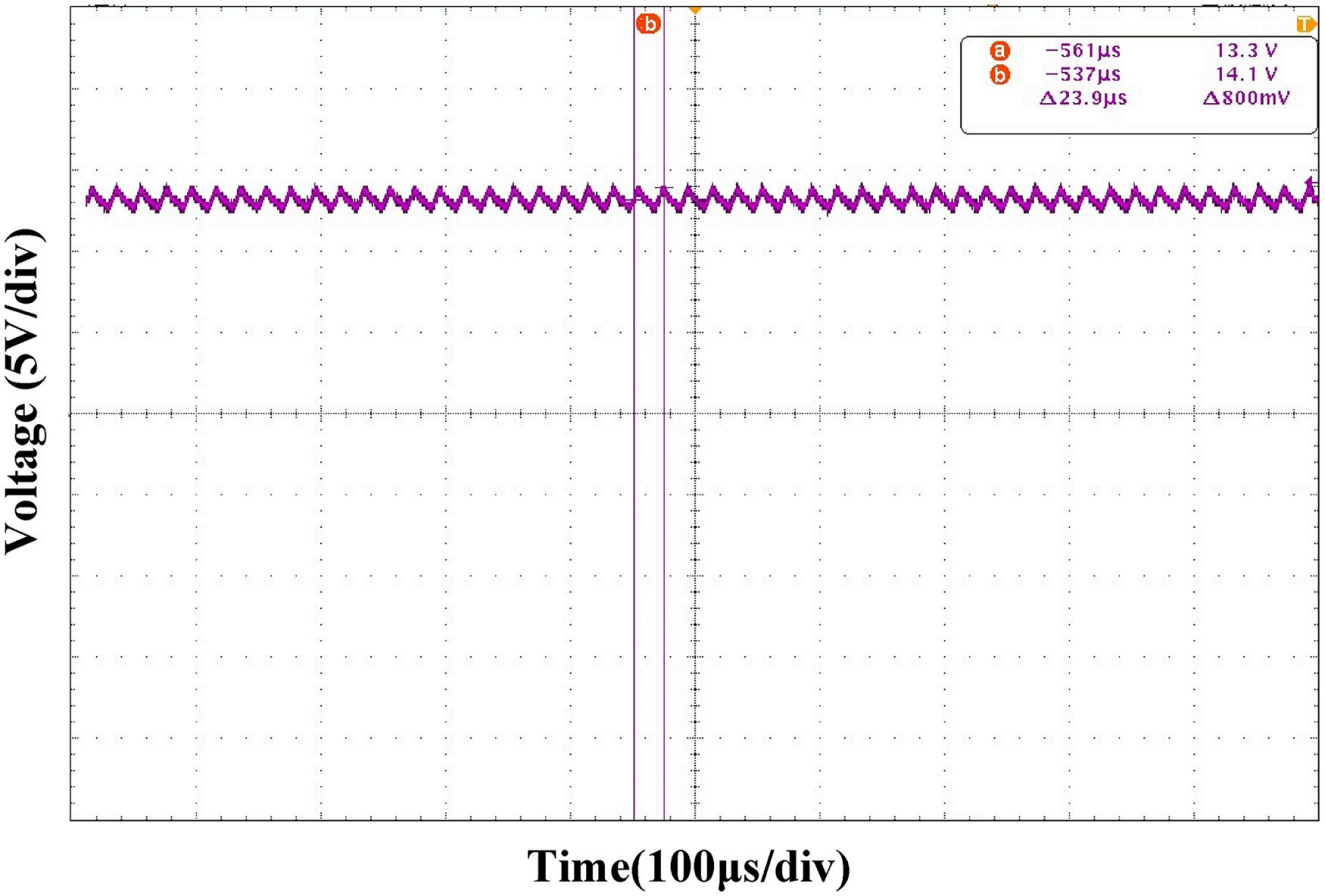
Output voltage waveform under PI control strategy.

**Fig 26:**
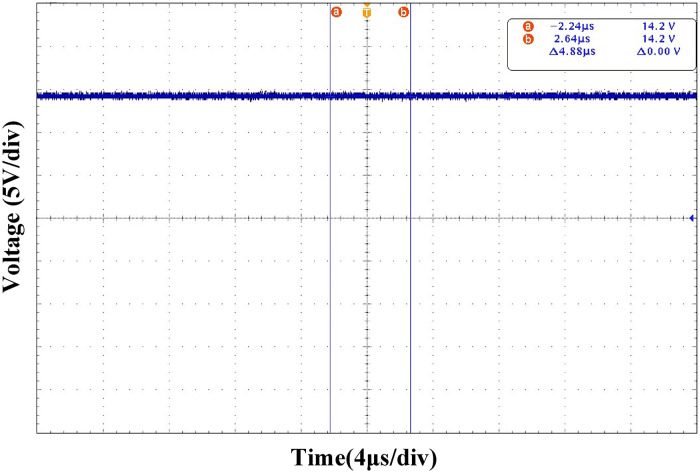
Output voltage waveform under ATSMC control strategy.

Figs 25 and 26 show the output voltage steady-state waveform under the PI and ATSMC control strategies, respectively. According to the output voltage waveform analysis, the output voltage ripple under the PI control strategy is 800mV, which is larger than that under the ATSMC control strategy. Therefore, the output voltage accuracy of the ATSMC strategy was higher.

**Fig 27:**
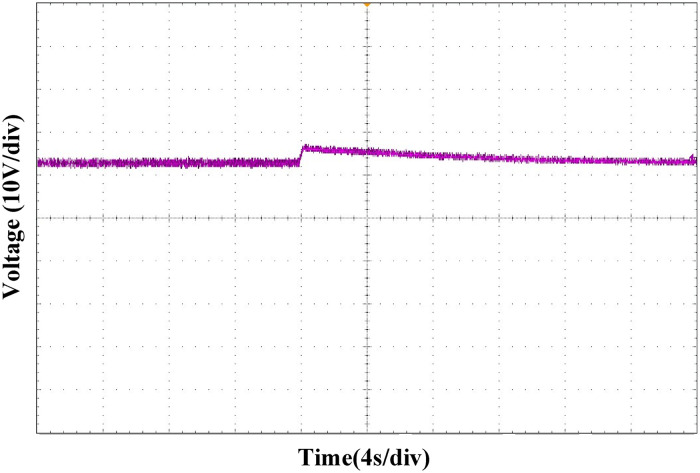
Load switching waveform under PI control strategy.

**Fig 28:**
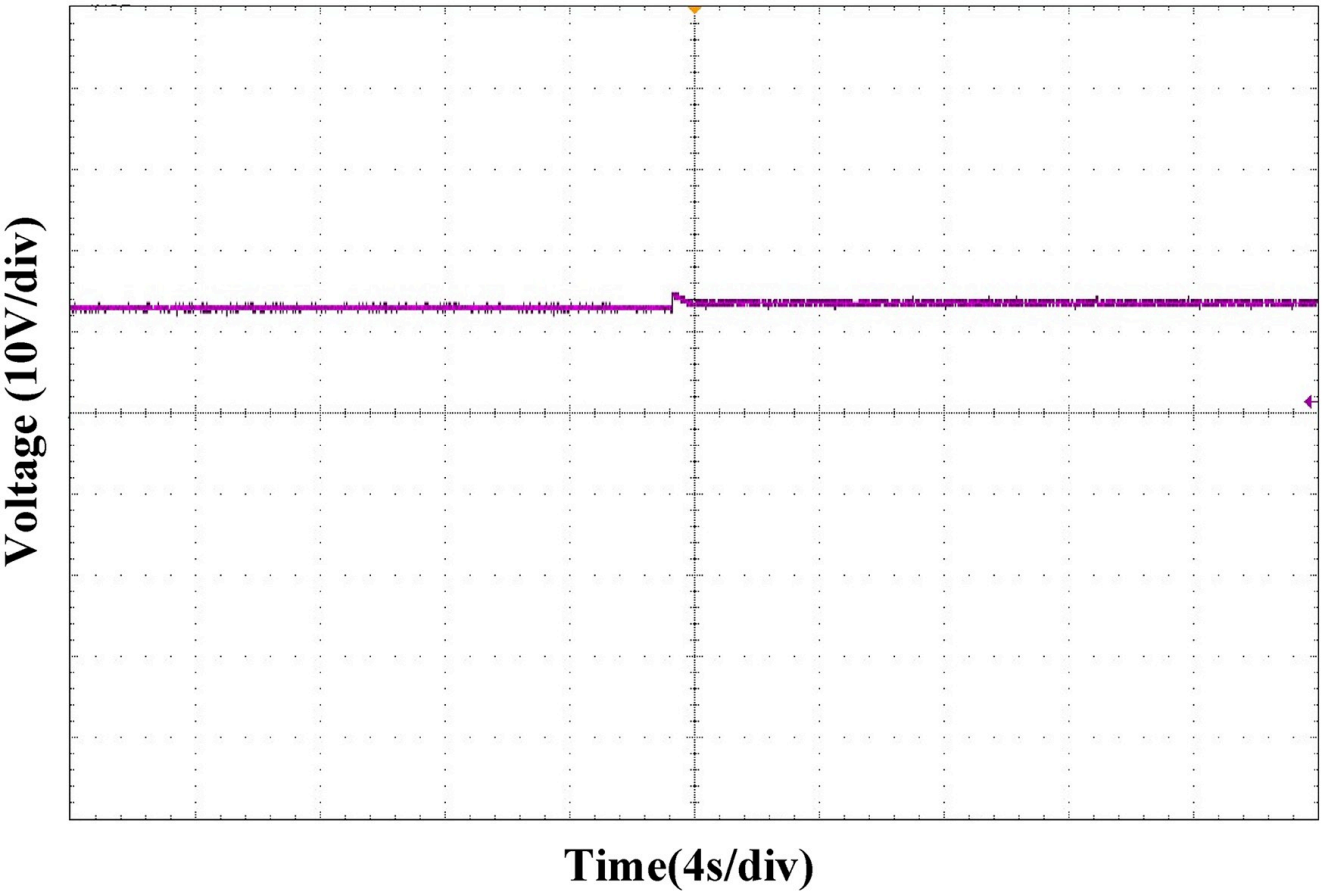
Load switching waveform under ATSMC control strategy.

**Fig 29:**
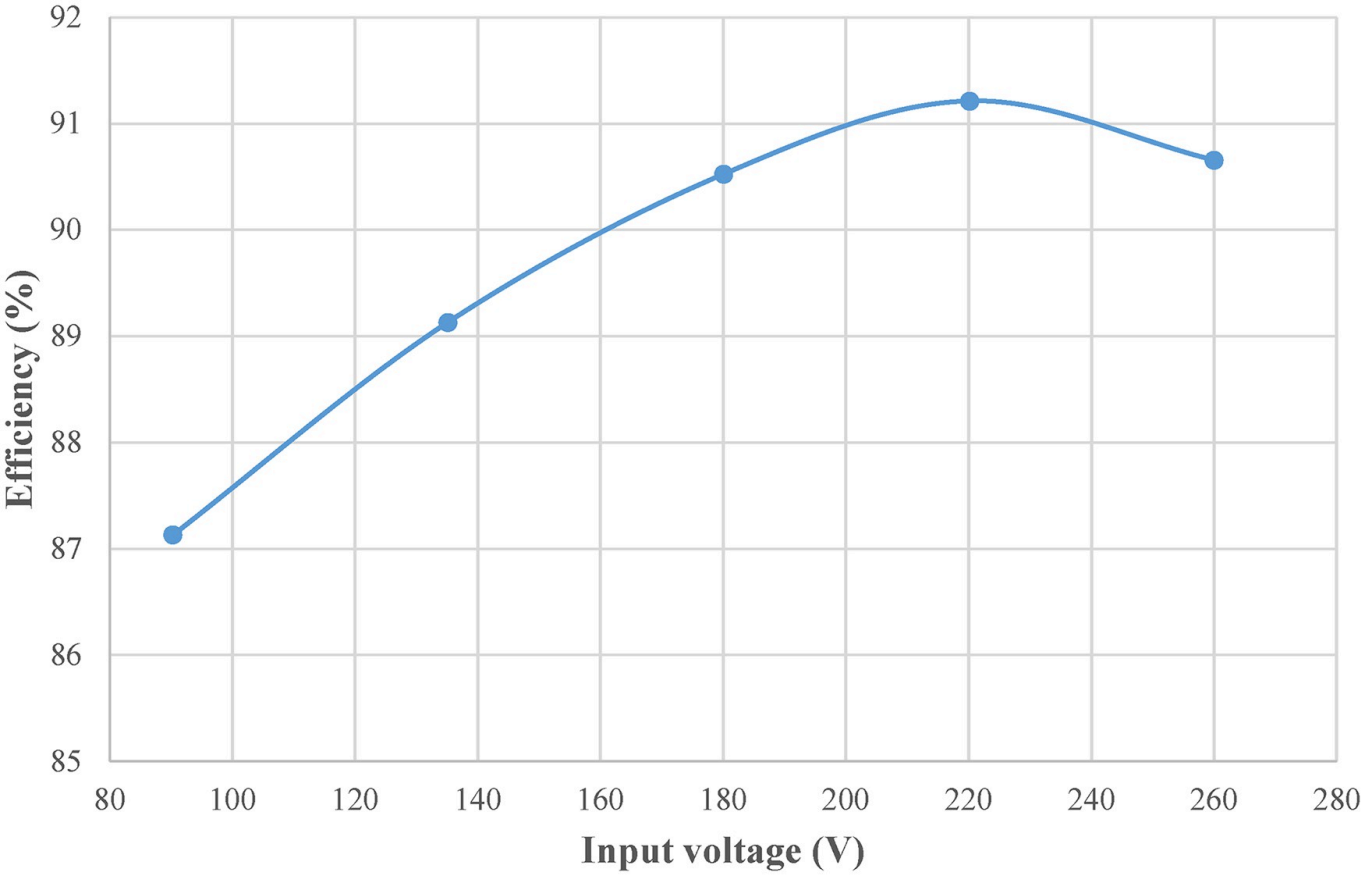
Efficiency curve of front-stage Buck PFC converter.

**Fig 30:**
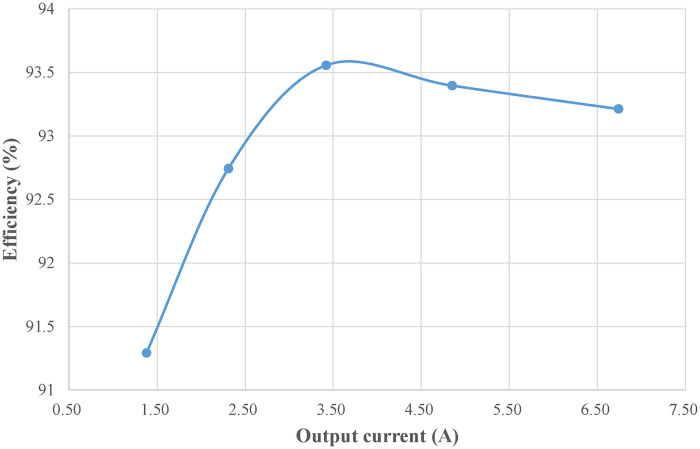
Efficiency curve of rear-stage full-bridge converter.

Figs 27 and 28 show the output voltage waveform comparison between the PI and ATSMC control strategies when the load is switched. Compared with PI control, when the same degree of load change occurred, under the ATSMC control strategy, the system adjustment time was shorter, the instantaneous voltage change was smaller, and the system robustness was stronger.

(Fig 29) shows the efficiency curve of the front-stage Buck PFC circuit under different input voltages. (Fig 30) shows the efficiency curves of the rear-stage full-bridge converter under different loads. Multiplying the efficiencies of the front and rear stages under rated conditions shows that the combined efficiency of the two-stage converter is above 85%.

## 6 Conclusion

In this study, a two-stage AC/DC converter was designed. The front stage uses a Buck PFC circuit operating in discontinuous capacitor voltage mode, and the rear-stage uses a full-bridge converter. The parameters of the circuit components were obtained through theoretical analysis and calculations. The PI control strategy and parameter adaptive terminal sliding mode control strategy were adopted for the front-stage and rear-stage circuits, respectively. The control effect was verified through simulation, and finally, a physical circuit was built. Experiments show that the circuit realized a power-factor correction. The corrected system power factor exceeded 90% under full-load conditions, and 98% under rated conditions. The rear-stage full-bridge circuit realized zero-voltage conduction of MOSFETs. Compared with traditional PI control, the response speed of the circuit startup phase was faster, and the output voltage accuracy was higher. When the load changes, the system exhibits a faster adjustment speed, less voltage fluctuation, and stronger system robustness.
